# Supplementary material for: Towards a better understanding of risk selection in maternal and newborn care: A systematic scoping review
Source: PLoS One. 2020 Jun 8;15(6):e0234252. doi: 10.1371/journal.pone.0234252 (PMC7279596; doi:10.1371/journal.pone.0234252)
Supplement: S2 Table — (DOCX) [file pone.0234252.s002.docx]

**S2 Search strategies**

**PubMed History April 16, 2019**

| **Search** | **PubMed Query** | **Items found** |
| --- | --- | --- |
| #5 | #1 AND #2 AND #3 AND #4 | 4756 |
| #4 | “Outcome Assessment (Health Care)”[Mesh] OR “Pregnancy Outcome”[Mesh] OR “Morbidity”[Mesh] OR “Mortality”[Mesh] OR “Medicalization”[Mesh] OR “Pregnancy Complications”[Mesh] OR “Obstetric Labor Complications”[Mesh] OR outcome*[tiab] OR morbidit*[tiab] OR mortalit*[tiab] OR death*[tiab] OR medicalization*[tiab] OR medicalisation*[tiab] OR overestimation[tiab] OR underestimation[tiab] OR complication*[tiab] | 4503119 |
| #3 | “Primary Health Care”[Mesh] OR “Family Practice”[Mesh] OR “Physicians, Primary Care”[Mesh] OR “General Practitioners”[Mesh] OR “Physicians, Family”[Mesh] OR “Midwifery”[Mesh] OR “Nurse Midwives”[Mesh] OR “Obstetrics”[Mesh] OR “Perinatal Care”[Mesh] OR “Prenatal Care”[Mesh] OR primary care[tiab] OR primary health care[tiab] OR primary healthcare[tiab] OR first line care[tiab] OR family practice[tiab] OR family physician*[tiab] OR general practitioner*[tiab] OR general practice physician*[tiab] OR gp[tiab] OR midwife*[tiab] OR midwiv*[tiab] OR birth attendant*[tiab] OR obstetrician[tiab] OR obstetrics[tiab] OR obstetrical care[tiab] OR gynaecolog*[tiab] OR gynecolog*[tiab] OR perinatal care[tiab] OR postnatal care[tiab] OR prenatal care[tiab] OR natal care[tiab] | 511260 |
| #2 | “Triage”[Mesh] OR “Gatekeeping”[Mesh] OR “Referral and Consultation”[Mesh:NoExp] OR “Patient Transfer”[Mesh] OR referral*[tiab] OR gatekeep*[tiab] OR triage*[tiab] OR transfer of care[tiab] OR risk selection[tiab] | 173907 |
| #1 | “Risk”[Mesh:NoExp] OR “Risk Factors”[Mesh] OR “Uncertainty”[Mesh] OR risk*[tiab] OR high chance*[tiab] OR higher chance*[tiab] OR high probabilit*[tiab] OR higher probabilit*[tiab] OR uncertain*[tiab] | 2436108 |

**Embase.com History April 16, 2019**

| **Search** | **Embase Query** | **Items found** |
| --- | --- | --- |
| #5 | #1 AND #2 AND #3 AND #4 | 5054 |
| #4 | 'treatment outcome'/exp OR 'pregnancy outcome'/exp OR 'morbidity'/exp OR 'mortality'/de OR 'infant mortality'/exp OR 'maternal mortality'/exp OR 'perinatal mortality'/exp OR 'prenatal mortality'/exp OR 'fetus mortality'/exp OR 'medicalization'/exp OR 'pregnancy complication'/exp OR 'labor complication'/exp OR outcome*:ab,ti,kw OR morbidit*:ab,ti,kw OR mortalit*:ab,ti,kw OR death*:ab,ti,kw OR medicalization*:ab,ti,kw OR medicalisation*:ab,ti,kw OR overestimation:ab,ti,kw OR underestimation:ab,ti,kw OR complication*:ab,ti,kw | 5619538 |
| #3 | 'primary health care'/exp OR 'general practice'/exp OR 'general practitioner'/exp OR 'midwife'/exp OR 'nurse midwife'/exp OR 'obstetrics'/exp OR 'perinatal care'/de OR 'prenatal care'/de OR 'postnatal care'/de OR ‘primary care’:ab,ti,kw OR ‘primary health care’:ab,ti,kw OR ‘primary healthcare’:ab,ti,kw OR ‘first line care’:ab,ti,kw OR ‘family practice’:ab,ti,kw OR ‘family physician*’:ab,ti,kw OR ‘general practitioner*’:ab,ti,kw OR ‘general practice physician*’:ab,ti,kw OR gp:ab,ti,kw OR midwife*:ab,ti,kw OR midwiv*:ab,ti,kw OR ‘birth attendant*’:ab,ti,kw OR obstetrican*:ab,ti,kw OR obstetrics:ab,ti,kw OR ‘obstetrical care’:ab,ti,kw OR gynaecolog*:ab,ti,kw OR gynecolog*:ab,ti,kw OR ‘perinatal care’:ab,ti,kw OR ‘postnatal care’:ab,ti,kw OR ‘prenatal care’:ab,ti,kw OR ‘natal care’:ab,ti,kw | 652243 |
| #2 | 'patient referral'/exp OR referral*:ab,ti,kw OR gatekeep*:ab,ti,kw OR triage*:ab,ti,kw OR ‘transfer of care’:ab,ti,kw OR ‘risk selection’:ab,ti,kw | 243689 |
| #1 | 'risk'/de OR 'risk factor'/exp OR 'uncertainty'/exp OR risk*:ab,ti,kw OR ‘high* chance*’:ab,ti,kw OR ‘high* probabilit*’:ab,ti,kw OR uncertain*:ab,ti,kw | 3343575 |

**Cinahl (Ebsco) History April 16, 2019**

| **Search** | **Cinahl (Ebsco) Query** | **Items found** |
| --- | --- | --- |
| S5 | S1 AND S2 AND S3 AND S4 | 1381 |
| S4 | MH (“Treatment Outcomes” OR “Pregnancy Outcomes” OR “Outcomes (Health Care)” OR “Morbidity” OR “Mortality” OR “Infant Mortality” OR “Maternal Mortality” OR “Perinatal Death” OR “Pregnancy Complications” OR “Obstetric Emergencies” OR “Labor Complications” OR “Fetal Diseases”) OR TI ( outcome* OR morbidit* OR mortalit* OR death* OR medicalization* OR medicalisation* OR overestimation OR underestimation OR complication* ) OR AB ( outcome* OR morbidit* OR mortalit* OR death* OR medicalization* OR medicalisation* OR overestimation OR underestimation OR complication* ) | 1032361 |
| S3 | MH (“Primary Health Care” OR “Physicians, Family” OR “Family Practice” OR “Midwives+” OR “Obstetrics” OR “Obstetric Care” OR “Perinatal Care” OR “Prenatal Care” OR “Postnatal Care”) OR TI (“primary care” OR “primary health care” OR “primary healthcare” OR “first line care” OR “family practice” OR “family physician*” OR “general practitioner*” OR “general practice physician*” OR gp OR midwife* OR midwiv* OR “birth attendant*” OR obstetrican* OR obstetrics OR “obstetrical care” OR gynaecolog* OR gynecolog* OR “perinatal care” OR “postnatal care” OR “prenatal care” OR “natal care” ) OR AB ( “primary care” OR “primary health care” OR “primary healthcare” OR “first line care” OR “family practice” OR “family physician*” OR “general practitioner*” OR “general practice physician*” OR gp OR midwife* OR midwiv* OR “birth attendant*” OR obstetrican* OR obstetrics OR “obstetrical care” OR gynaecolog* OR gynecolog* OR “perinatal care” OR “postnatal care” OR “prenatal care” OR “natal care” ) | 208525 |
| S2 | MH (“Referral and Consultation” OR “Gatekeeping” OR “Triage” OR “Transfer, Discharge”) OR TI (referral* OR gatekeep* OR triage* OR “transfer of care” OR “risk selection” ) OR AB (referral* OR gatekeep* OR triage* OR “transfer of care” OR “risk selection”) | 76920 |
| S1 | MH (“Risk Factors” OR “Uncertainty”) OR TI (risk* OR “high* chance*” OR “high* probabilit*” OR uncertain*) OR AB (risk* OR “high* chance*” OR “high* probabilit*” OR uncertain*) | 667030 |

**Cochrane Library History April 16, 2019**

| **Search** | **Cochrane Library Query** | **Items found** |
| --- | --- | --- |
| #5 | #1 AND #2 AND #3 AND #4 | 1010 |
| #4 | (outcome* OR morbidit* OR mortalit* OR death* OR medicalization* OR medicalisation* OR overestimation OR underestimation OR complication*):ti,ab,kw (Word variations have been searched | 631126 |
| #3 | (“primary care” OR “primary health care” OR “primary healthcare” OR “first line care” OR “family practice” OR family NEXT physician* OR general NEXT practitioner* OR general NEXT practice NEXT physician* OR gp OR midwife* OR midwiv* OR birth NEXT attendant* OR obstetrician OR obstetrics OR “obstetrical care” OR gynaecolog* OR gynecolog* OR “perinatal care” OR “postnatal care” OR “prenatal care” OR “natal care”):ti,ab,kw (Word variations have been searched | 50869 |
| #2 | (referral* OR gatekeep* OR triage* OR transfer* OR “risk selection”):ti,ab,kw (Word variations have been searched | 32202 |
| #1 | (risk* OR high* NEXT chance* OR high* NEXT probabilit* OR uncertain*):ti,ab,kw (Word variations have been searched) | 221918 |
