# Supplementary material for: Towards a better understanding of risk selection in maternal and newborn care: A systematic scoping review
Source: PLoS One. 2020 Jun 8;15(6):e0234252. doi: 10.1371/journal.pone.0234252 (PMC7279596; doi:10.1371/journal.pone.0234252)
Supplement: S3 Table — (DOCX) [file pone.0234252.s003.docx]

**S3 Overarching themes, main categories and sub categories**

| Regulation  Provider centred focus  Avoiding underuse | | |
| --- | --- | --- |
| Aligning risk and resources   - Availability of care - Timeliness of care - Access to care   - Geographical regulation   - Medical regulation   - Financial regulation | Detecting risk, assessing risk and decision-making   - Tools - Professionals - Mothers - Healthcare organisation - Division of tasks and responsibilities   - Multidisciplinary agreements - Timing | Ensuring safety   - Supervision - Optimal/effective   - Morbidity/mortality   - Intervention rate   - Referral rate   - Comparison   - Professional decision making   - Mothers’ experiences |
