# Supplementary material for: Towards a better understanding of risk selection in maternal and newborn care: A systematic scoping review
Source: PLoS One. 2020 Jun 8;15(6):e0234252. doi: 10.1371/journal.pone.0234252 (PMC7279596; doi:10.1371/journal.pone.0234252)
Supplement: S4 Table — (DOCX) [file pone.0234252.s004.docx]

**S4 Characteristics of included references**

| **Reference ID** | **First author** | **Year of publication** | **Country**  Country studied. If not applicable, country first author | **Objective** | **Design** | **Dominant category** |
| --- | --- | --- | --- | --- | --- | --- |
| [1]* | Aalfs | 2003 | Netherlands | To explore the role of the general practitioner in referring women for genetic counselling during, instead of before a pregnancy | Questionnaire | Detecting risk, assessing risk and decision-making |
| [2]* | Accortt | 2017 | USA | To examine theory and research on mood and anxiety disorders during the  perinatal period with an emphasis on screening recommendations | Literature review | Detecting risk, assessing risk and decision-making |
| [3]* | Allen | 2007 | Ireland | To identify possible mechanisms by which caseload midwifery reduces preterm birth for young childbearing women | Mixed methods consisting of a cohort study and an ethnographic study | Aligning risk and resources |
| [4]* | Amelink-Verburg | 2008 | Netherlands | To assess the nature and outcome of intrapartum referrals from primary to secondary care within the obstetric system | Retrospective cohort study using a case database | Ensuring safety |
| [5]* | Amelink-Verburg | 2009 | Netherlands | To assess the trends and patterns of referral from midwives to obstetricians, and the differences in referral patterns between nulliparous and parous women | Retrospective cohort study using a case database | Ensuring safety |
| [6]* | Amelink-Verburg | 2010 | Netherlands | To study the evolution of the concept of ‘‘normality’’ by comparing the development and the contents of the consecutive versions of guidelines | Review of guidelines | Detecting risk, assessing risk and decision-making |
| [7]* | Ammari | 1995 | England | To study the adherence to the locally-agreed policy of screening by clinical risk factors | Retrospective cohort study using a case records | Detecting risk, assessing risk and decision-making |
| [8]* | Anderson | 1995 | USA | To describe the outcomes of planned home births attended by certified nurse-midwives | Retrospective survey | Ensuring safety |
| [9]* | Badgery-Parker | 2012 | Australia | To assess the frequency and outcomes of preterm hospital  admissions during pregnancy, with a focus on transfers to higher levels of care | Population-based cohort study using linked population data | Aligning risk and resources |
| [10]* | Bahry | 1989 | USA | To present the implementation of the university-based programme of perinatal care, centered on the use of a multidisciplinary health care team and emphasizing a decentralized service model with central referral and management of high risk problems | Descriptive retrospective cohort study | Aligning risk and resources |
| [11]* | Baird | 1996 | Scotland | To evaluate the use of a maternity  unit run by general practitioners and midwives, describing the outcome of labor in an unselected group of women and quantifying the contribution made by general practitioners | Retrospective population based review of case records | Ensuring safety |
| [12]* | Bais | 2004 | Netherlands | To evaluate the performance of abdominal palpation as a screening test for intrauterine growth retardation in a low risk population, under standard practice conditions | Retrospective population based observational study | Detecting risk, assessing risk and decision-making |
| [13]* | Baker | 1992 | USA | To describe the effect of a project, directing physicians to screen all Medicaid-eligible pregnant women and newborns for specified clinical high risk factors | Retrospective review using a database and case records | Aligning risk and resources |
| [14]* | Baldwin | 1995 | USA | To test the hypothesis that physicians with greater malpractice claims exposure, either through personal experience or in their practice environment, will use more prenatal resources and have a higher caesarean delivery rate than physicians with lesser claims exposure | Retrospective cohort study using a database data and case records | Detecting risk, assessing risk and decision-making |
| [15] | Baldwin | 2019 | Australia | To explored the transitioning of care models | Literature review | Aligning risk and resources |
| [16] | Bernitz | 2011 | Norway | To investigate possible differences in operative delivery rate among low-risk women, randomized to an alongside  midwifery-led unit or to standard obstetric units within the same hospital | Randomized controlled trial | Ensuring safety |
| [17] | Bernstein | 2016 | USA | To identify barriers and facilitators to testing and referral from patient and providers’ perspectives | Interviews | Detecting risk, assessing risk and decision-making |
| [18]* | Binders | 2011 | USA | To test the hypothesis that the promotion of national guidelines  recommending the transfer of high-risk mothers to subspecialty  perinatal centers reduces mortality and morbidity through the reduction  of preterm infants delivered at non-tertiary maternity hospitals | A population based cohort study | Detecting risk, assessing risk and decision-making |
| [19]* | Biro | 1991 | Australia | To review the safety of team midwifery care, in terms of perinatal mortality, for pregnant women assessed at their first visit as being at low risk of complications | Retrospective cohort study | Ensuring safety |
| [20]* | Blix | 2016 | Norway, Sweden, Denmark and Iceland | To describe the indications for transfer to hospital in planned home births, and the proportion of cases in which this occurs | Prospective cohort study | Ensuring safety |
| [21] | Blondel | 2009 | 9 European countries  Belgium, Denmark, France, Germany, Italy, Netherlands, Poland, Portugal and UK (England, Ireland, Scotland, Wales) | To study the impact of the organization of obstetric services on the regionalization of care for very preterm  births | Retrospective cohort study | Ensuring safety |
| [22] | Bode | 2001 | USA | To analyze trends across time in the regionalization of low-birth-weight births and time trends for the association between regionalization and decreased neonatal mortality | Retrospective cohort study | Aligning risk and resources |
| [23] | Bodner | 2017 | Austria | To analyze the maternal and neonatal outcomes of a midwife-led care service was incorporated within the department of obstetrics and fetomaternal medicine | Retrospective cohort study | Ensuring safety |
| [24] | Bolbocean | 2016 | Canada | To estimate the effect of highest levels of neonatal care available at delivery on the risk of developing a non-ambulatory cerebral palsy status | Retrospective cohort study | Ensuring safety |
| [25] | Bovbjerg | 2017 | USA | To examine the independent contributions of a variety of risk factors to birth outcomes among women planning community birth | Retrospective cohort study | Detecting risk, assessing risk and decision-making |
| [26]* | Britt | 2006 | USA | To evaluate the joint impact of pregnancy risk and the timing of referral of high-risk pregnancies from obstetricians to maternal fetal medicine sub-specialists on gestational age at delivery | Retrospective cohort study | Detecting risk, assessing risk and decision-making |
| [27] | Bronstein | 2011 | USA | To examine the factors associated with delivery of preterm infants at neonatal intensive care unit hospitals | Retrospective cohort study using two databases | Detecting risk, assessing risk and decision-making |
| [28] | Butler | 2015 | Ireland | To evaluate midwife-led care antenatal care compared with antenatal care provided in traditional obstetric-led hospital antenatal clinics | Mixed methods consisting of a chart audit, a postal survey, a focus group and interviews | Ensuring safety |
| [29]* | Carolan | 2009 | Canada | To explore women's experiences of referral to tertiary care on the basis of uncertain ultrasound findings | Interviews | Ensuring safety |
| [30] | Chambliss | 1992 | USA | To test the hypothesis that the low caesarean birth rate on the midwifery service was a result of patient selection bias | Randomized blinded clinical trial | Ensuring safety |
| [31]* | Cohen | 2015 | France | To assess the knowledge and clinical  attitude of obstetricians and/or gynecologists regarding  periodontal diseases | Cross-sectional study using a questionnaire | Detecting risk, assessing risk and decision-making |
| [32] | Cooke | 2004 | Australia | To demonstrate the effectiveness of evidence based models and systems of care on improving patient outcomes | Mixed methods using a literature review, a survey and a panel | Aligning risk and resources |
| [33]* | Cordero | 1989 | USA | To study the appropriateness of antenatal referrals for patients at risk for premature delivery | Retrospective cohort study using case records | Ensuring safety |
| [34]* | Craig | 1985 | USA | To examine the use of obstetrical  consultants by family medicine residents and faculty | Retrospective cohort study using case records | Ensuring safety |
| [35] | Crotty | 1990 | Australia | To describe homebirths and indicate aspects that require special attention | Retrospective cohort study using case records | Ensuring safety |
| [36]* | Daemers | 2014 | Netherlands | To assess the impact of obesity on the likelihood of remaining in midwife-led care throughout pregnancy and childbirth | Prospective cohort study | Detecting risk, assessing risk and decision-making |
| [37]* | David | 2006 | Germany | Investigation of the reasons for the transfer of women from a birth center to a hospital in the course of childbirth as well as modalities and effects | Prospective cohort study using a questionnaire | Ensuring safety |
| [38]* | De Galan-Roosen | 1999 | Netherlands | To establish the distribution of perinatal mortality over the various levels of obstetric care, taking into account the various causes of perinatal mortality | Prospective cohort study using a case database | Ensuring safety |
| [39] | De Jonge | 2009 | Netherlands | To compare perinatal mortality and severe perinatal morbidity between planned home and planned hospital births, among low-risk women who started their labor in primary care | Retrospective cohort study using a case database | Ensuring safety |
| [40]* | De Jonge | 2011 | Netherlands | To assess whether midwives adjust their care if women are undocumented and have no health insurance | Retrospective matched cohort study | Detecting risk, assessing risk and decision-making |
| [41] | De Jonge | 2013 | Netherlands | To test the hypothesis that low risk women at the onset of labor with planned home birth have a higher rate of severe acute maternal morbidity than women with planned hospital birth, and to compare the rate of postpartum hemorrhage and manual removal of placenta | Retrospective cohort study using a linked database | Ensuring safety |
| [42]* | De Jonge | 2015 | Netherlands | To test the hypothesis that it is possible to select a group of low risk women who can start labor in midwife-led care without having increased rates of severe adverse maternal outcomes compared to women who start labor in secondary care | Retrospective cohort study using a case database | Ensuring safety |
| [43]* | De Reu | 2010 | Netherlands | To analyze avoidable perinatal mortality in small-for-gestational-age children | Retrospective study using a case database | Ensuring safety |
| [44]* | De Weerd | 2001 | Netherlands | To provide an overview of the outcomes from an outpatients' clinic for preconception counselling i and to assess its activities in terms of referring professionals, referral indications, supplementary investigations, counselling and treatment policy | Retrospective study using case records | Ensuring safety |
| [45]* | DeJong | 1981 | USA | To presents data on patient characteristics, birth outcomes of out-of-hospital delivery and freestanding midwifery group | Retrospective review of case record | Ensuring safety |
| [46]* | Delaney-Black | 1989 | USA | To evaluated antepartum maternal and postnatal infant referrals from five metropolitan hospitals with level I facilities | Retrospective review of delivery room logs and case records | Detecting risk, assessing risk and decision-making |
| [47] | Dencker | 2017 | Ireland | To evaluate maternal and neonatal  outcomes and transfer rates during six years of midwife led unite sites | Retrospective cohort study using case records | Ensuring safety |
| [48]* | Dijkstra | 2003 | Netherlands | To study the management and views regarding threatened preterm delivery prevalent in the three professions providing obstetric care | Questionnaire | Detecting risk, assessing risk and decision-making |
| [49] | Donohue | 2009 | USA | To investigate prenatal management and outcome of infants born at the border of viability during two periods | Retrospective cohort study using case records | Ensuring safety |
| [50]* | Eden | 2005 | USA | To assess the effect of sub-specialty prenatal care provided to high-risk obstetrical patients in a community perinatal center as a function of whether consultation and referral to a maternal–fetal medicine sub-specialist was at the discretion of the generalist, required by the insurance carrier or by patient choice | Retrospective cohort study using case records | Aligning risk and resources |
| [51] | Eide | 2009 | Norway | To compare intervention rates associated with labor in low-risk women who begin their labor in a midwife-led unit and a conventional care unit | Prospective cohort study using case records | Ensuring safety |
| [52]* | Engjom | 2018 | Norway | To examine the association between availability of obstetric institutions and risk of eclampsia, HELLP-syndrome, or delivery before 35 gestational weeks in pre-eclamptic pregnancies | National population-based retrospective cohort study | Aligning risk and resources |
| [53] | Eskes | 2017 | Netherlands | To assess the underlying risk factors for perinatal mortality in term born small for gestational age infants | Retrospective cohort study using case records | Detecting risk, assessing risk and decision-making |
| [54]* | Evers | 2010 | Netherlands | Compare incidences of perinatal mortality and severe perinatal morbidity between low risk term pregnancies supervised in primary care by a midwife and high risk pregnancies supervised in secondary care by an obstetrician | Prospective cohort study using case records | Ensuring safety |
| [55]* | Evers | 2013 | Netherlands | To assess substandard care factors in the case of delivery-related asphyxia | Prospective cohort study using case records | Ensuring safety |
| [56] | Ferndale | 2017 | Australia | To examine how risk shapes interactions between midwives and pregnant women in the context of public hospitals | Recordings of consultations | Detecting risk, assessing risk and decision-making |
| [57]* | Ferrazzi | 2015 | Italy | To analyze maternal and neonatal outcomes of midwife-led labor in low-risk women at term | Prospective cohort study | Ensuring safety |
| [58]* | Finnström | 2006 | Sweden | To evaluate the quality of perinatal care in relation to size of delivery unit and size of catchment area for deliveries to evaluate referral system high risk pregnancies | Retrospective cohort study using two case records databases | Ensuring safety |
| [59] | Fleissig | 1996 | England | To assess the feasibility of obstetric offering community-led maternity care to most women, both those assessed to be at low obstetric risk and those with complicated pregnancies at 'booking' | Retrospective cohort study using case records | Ensuring safety |
| [60] | Ford | 1991 | England | To assess the outcome of pregnancy  for women booking for home births | Retrospective review of case records | Ensuring safety |
| [61]* | Fullerton | 1997 | USA | To study the applicability of transfer rates from two freestanding birth centers as a critical clinical indicator | Prospective cohort study using aggregated data summery reports and clinical logs | Ensuring safety |
| [62]* | Garite | 1995 | USA | To describe our experience with a freestanding birthing center established in conjunction with a university medical center, and to determine the safety and effectiveness of such a program | Prospective using a database | Ensuring safety |
| [63] | Gaudineau | 2013 | France | To compare the intervention rates associated with labor in low-risk women who began their labor in the home-like birth center and the traditional labor ward | Retrospective study using case records | Ensuring safety |
| [64]* | Geerts | 2014 | Netherlands | To compare sense of control, which is a major attribute of the childbirth experience, for women planning home compared to women planning hospital birth under midwife-led care | Retrospective cohort study using a database | Ensuring safety |
| [65] | George | 2018 | Australia | To assess the effectiveness of a midwifery-initiated oral health dental service program in improving uptake of dental services, oral health knowledge, quality of oral health, oral health status and birth outcomes of pregnant women | Multi-center randomized controlled trial | Ensuring safety |
| [66]* | Giles | 2000 | Australia | To determine whether the introduction of routine fetal fibronectin bedside testing affected costs and transfer rates from referral district hospitals to a tertiary obstetric hospital, as well as direct admissions to a tertiary referral hospital | Prospective audit of cases | Detecting risk, assessing risk and decision-making |
| [67] | Gillespie | 2018 | Ireland | To identify the nature, content and accessibility of educational resources available to health professionals caring for  pregnant women with heart disease | Scoping literature review | Detecting risk, assessing risk and decision-making |
| [68]* | Godbole | 2013 | England | To assess adherence to the national guidelines for management of Hepatitis B infection in pregnancy | Retrospective audit using case records and discharge summaries | Detecting risk, assessing risk and decision-making |
| [69]* | Goh | 2015 | Australia | To document the outcomes of high-risk obstetric transfers | Prospective observational study of cases | Ensuring safety |
| [70] | Govaerts | 2017 | Netherlands | To evaluate the pregnancy outcomes in susceptibility loci cases and to establish a protocol for pregnancy management, follow-up and additional investigations | Case evaluation | Detecting risk, assessing risk and decision-making |
| [71] | Grigg | 2015 | New Zealand | To examine the transfers from primary maternity units to a tertiary hospital by describing the frequency, timing, reasons and outcomes of those who had antenatal or pre-admission birthplace plan changes, and transfers in labor or postnatally | Mixed methods prospective cohort study using survey data and transfer data | Aligning risk and resources |
| [72] | Gyte | 2009 | England and Wales | To critically appraise a study on the safety of home birth (Mori R, Dougherty M, Whittle M. BJOG 2008;115:554) and assess its contribution to the debate about risks and benefits of planned home birth for women at low risk of complications | Critical appraisal of a published paper | Ensuring safety |
| [73]* | Hein | 1986 | USA | To describe the Iowa regional perinatal care system and to offer evaluation of the effects of this system on perinatal outcome in the state | Retrospective cohort study using a database | Aligning risk and resources |
| [74] | Hemmeniki | 1990 | Finland | To describe the use of the clinics and to see whether clinics serve as an addition or as an alternative to maternity centers | Mixed methods using case records, interviews and a questionnaire | Aligning risk and resources |
| [75] | Hemminiki | 2011 | Finland | To describe trends in centralization and unplanned out-of-hospital births, perinatal mortality by place of birth, and health and birth outcomes in areas served by hospitals of different levels | Cross-sectional study using a database | Ensuring safety |
| [76]* | Hollingworth | 2018 | Australia | To describe the outcomes of patients transferred to a hospital with signs of labor at preterm gestations | Retrospective observational study using cases | Detecting risk, assessing risk and decision-making |
| [77]* | Holt | 2001 | Norway | To report a two year study from this isolated rural hospital with the aim of estimating the feasibility and the effectiveness of the case selection process | Prospective cohort study using case records | Aligning risk and resources |
| [78] | Hopkins | 2018 | USA | To define the most common forms of congenital heart disease in pregnancy, outline preconception counselling, discuss the associated morbidity and mortality of each lesion, and review current recommendations  for management of congenital heart disease in pregnancy | Literature review | Detecting risk, assessing risk and decision-making |
| [79]* | Hueston | 1994 | USA | To examine referral patterns of family physicians who perform obstetrics to determine the effects of referral bias on family physician and obstetrician patient populations | Retrospective review of case records | Detecting risk, assessing risk and decision-making |
| [80] | Humphrey | 2017 | Australia | To review the usefulness of the revised pregnancy risk score system and  the integrity of its continuing use | Prospective study using risk score cards | Detecting risk, assessing risk and decision-making |
| [81] | Hundley | 1994 | Scotland | To examine whether intrapartum care and delivery of low risk women in a midwife managed delivery unit differs from that in a consultant led labor ward | Randomized controlled trial | Ensuring safety |
| [82]* | Hutchinson | 2014 | Australia | To determine, in women transferred antenatally for acute admission with high risk pregnancies, the numbers who deliver, the average time from transfer to delivery, and whether the reason for transfer influences the time-to-delivery | Retrospective study using case records | Aligning risk and resources |
| [83] | Hutton | 2009 | Canada | To compare maternal and perinatal/neonatal mortality and morbidity and intrapartum intervention rates for women attended by midwives who planned a home birth compared with similar low-risk women who planned a hospital birth | Retrospective cohort study using a database | Ensuring safety |
| [84]* | Jackson | 2006 | England | To explore women’s views on being referred to and attending a specialist antenatal hypertension clinic | Interviews | Ensuring safety |
| [85] | James | 2017 | UK (England, Ireland, Scotland, Wales) | To determine what constitutes high dependency care in obstetric units remote from tertiary referral centers | Delphi study | Detecting risk, assessing risk and decision-making |
| [86] | Janssen | 2003 | Canada | To evaluate accountability among midwives, defined as profession of safe and appropriate care and maintenance of standards of communication | Case records | Ensuring safety |
| [87] | Jeffery | 2017 | England | To explore midwives’ experiences of performing maternal observations | Focus groups | Detecting risk, assessing risk and decision-making |
| [88]* | Jordan | 1995 | Canada | To determine referral rates, to study the nature of consultations with obstetricians, and to examine how both patient and physician characteristics affect referrals | Retrospective review of case records | Detecting risk, assessing risk and decision-making |
| [89]* | Kim | 2010 | USA | To examine mental health referrals outcomes among obstetric patients at risk for depression | Interviews | Aligning risk and resources |
| [90]* | Kirke | 2010 | Australia | To explores how safe is rural general practitioner obstetrics | Retrospective review of case records | Ensuring safety |
| [91] | Knight | 2018 | UK (England, Ireland, Scotland, Wales) | To review the care of women with severe morbidity during or shortly after pregnancy in addition to those who die | Document review | Detecting risk, assessing risk and decision-making |
| [92]* | Knox | 1984 | USA | To explore how and under what circumstances to transport in-utero | Retrospective review of case records | Aligning risk and resources |
| [93] | Kollée | 1998 | Netherlands | To determine changes in referral of preterm newborns | Retrospective cohort study using a case database | Ensuring safety |
| [94]* | Koshida | 2015 | Japan | To investigate stillbirth, and to classify the possibilities of preventable stillbirths and collected  recommendations for prevention | Retrospective review of case records | Ensuring safety |
| [95]* | Kruske | 2015 | Australia | To describe the reasons for transfer to and from the unit, transfer times and the clinical health outcomes of all women and their babies | Retrospective review of audit data and case records | Ensuring safety |
| [96]* | Kruske | 2016 | Australia | To confirm and describe how primary maternity care units operate | Cross- sectional study using a survey | Aligning risk and resources |
| [97]* | Kuliukas | 2015 | Australia | To describe the overall labor and birth experience of partners within the context of an intrapartum transfer occurring from a low risk midwifery-led, woman-centered unit to an obstetric unit | Interviews | Ensuring safety |
| [98]* | Kwong | 2018 | USA | To evaluate physician practices and knowledge regarding Hepatitis B in women diagnosed perinatally | Survey | Detecting risk, assessing risk and decision-making |
| [99] | Lagendijk | 2018 | Netherlands | To investigate whether a combination of risk assessment focused on non-medical risk factors, lifestyle factors, and medical risk factors, with subsequent institution of risk-specific care pathways, and multidisciplinary consultation between care providers from the curative and the public health sector reduced adverse pregnancy outcomes | Cluster randomized controlled trial | Detecting risk, assessing risk and decision-making |
| [100]* | Lalor | 2007 | Ireland | To explore women’s experiences of encounters with caregivers after the diagnosis of fetal anomaly at the routine second trimester ultrasound scan | Interviews | Ensuring safety |
| [101]* | Laube | 1983 | USA | To describe the experiences with an alternative birth center | Retrospective review of case records | Ensuring safety |
| [102]* | Lavender | 2006 | England | To assess the effect of different action line positioning on birth outcomes | Randomized trial | Detecting risk, assessing risk and decision-making |
| [103]* | Law | 1999 | China | To compare the efficacy of midwife-managed care and obstetrician-managed care for women assessed to be at low risk in the initial intraparturn period | Prospective randomized controlled trial | Ensuring safety |
| [104]* | Leddy | 2011 | USA | To summarize studies that encompasses depression, anxiety, and eating disorders | Document review | Detecting risk, assessing risk and decision-making |
| [105]* | Lennox | 1992 | Scotland | To study in-utero and neonatal transfers from the perspective of transferring hospitals | Retrospective review of case records | Ensuring safety |
| [106]* | Lessaris | 2002 | USA | To determine whether perinatal referral patterns and clinical outcomes for very low birthweight infants changed in relation to changing Medicaid financial policies | Retrospective cohort using case records | Aligning risk and resources |
| [107] | Lim | 2017 | England | To compare the care delivered in a joint cardiac obstetric service against recognized international standards | Retrospective review of case records | Detecting risk, assessing risk and decision-making |
| [108]* | Lubchenco | 1989 | USA | To evaluate the effect of aggressive intrapartum and early neonatal resuscitation on perinatal mortality, neonatal morbidity, and long-term outcome | Retrospective cohort using case records | Ensuring safety |
| [109] | Lundeen | 2016 | USA | To describe the reasons for and outcomes of maternal transfers from private homes and freestanding birthing suites to a large academic hospital | Retrospective cohort using case records | Ensuring safety |
| [110] | Maassen | 2008 | Netherlands | To compare planned place of birth and incidence of operative delivery among women at low risk of complications at the time of onset of labor | Retrospective cohort study using a case database | Ensuring safety |
| [111] | MacVicar | 1993 | England | To compare the outcome of two methods of maternity care during the antenatal period and at delivery | Randomized controlled trial | Ensuring safety |
| [112]* | Magann | 2012 | USA | To determine maternal fetal medicine referral trends in a Medicaid  population over time | Retrospective study using a database | Detecting risk, assessing risk and decision-making |
| [113]* | Mansbridge | 2014 | England | To describe the development, testing, evaluation and implementation of a nurse-to-nurse referral pathway between two trusts | Retrospective review of case records | Ensuring safety |
| [114]* | Marlow | 2014 | England | To evaluate the hypothesis that birth in, or transfer to, designated referral centers is accompanied by lower rates of mortality and morbidity among survivors | Retrospective review of case records | Ensuring safety |
| [115]* | Martijn | 2013 | Netherlands | To analyze the determinants of risk of critical incidents for women with a low risk profile at the start of pregnancy with a view on improving patient safety | Retrospective review of case records | Ensuring safety |
| [116] | Matthey | 2016 | Scotland | To report the evaluation of the change in cut-off score in the Edinburgh Depression Scale clinical practice | Mixed methods using review of case records and interviews | Detecting risk, assessing risk and decision-making |
| [117]* | Mayer | 2018 | England | To describe the composition and processes of multidisciplinary care between maternity and cardiac services before, during and after pregnancy for women with cardiac disease, and explore clinicians’ (cardiologists, obstetricians, nurses, midwives) and women’s experiences of delivering/receiving care within these models | Mixed-methods comprising case-note audit, interviews and observations | Detecting risk, assessing risk and decision-making |
| [118] | McCool | 2015 | USA | To evaluate claims brought against midwives, with the intent of developing strategies to decrease the incidence of litigation | Retrospective review of case records | Ensuring safety |
| [119] | McIntyre | 2012 | Australia | To determine whether there is convincing evidence to support the safety of non-medically led models of primary maternity care | Literature review | Ensuring safety |
| [120]* | McMurtrie | 2009 | Australia | To report the outcomes of a homebirth programme | Prospective review of cases | Ensuring safety |
| [121] | Mengel | 1987 | USA | To determine whether family physicians are as competent in proving obstetric care as obstetricians | Literature review | Ensuring safety |
| [122] | Meuli | 1984 | USA | To describe process and success factors of implementation of regionalization | Retrospective review of case records | Aligning risk and resources |
| [123] | Mito | 2015 | Japan | To assess the present status of clinical care for postpartum patients with hypertensive disorders of pregnancy | Survey | Detecting risk, assessing risk and decision-making |
| [124] | Monk | 2014 | Australia | To compare maternal and neonatal birth outcomes and morbidities associated with the intention to give birth in two freestanding midwifery units and two tertiary-level maternity units | Prospective cohort study using case records | Ensuring safety |
| [125]* | Montgomery-Andersen | 2010 | Greenland | To document how women because of at-risk pregnancies narratively constructed self-understanding and defined meaning during their period of separation from family and community; and how they dealt with the challenges they were presented with | Interviews | Ensuring safety |
| [126]* | Morano | 2007 | Italy | To assess the experience a midwife-led birth center | Prospective review of cases | Ensuring safety |
| [127] | Morley | 2018 | England | To explore the complexities of care provision for women with epilepsy and includes recommendations to optimize maternity health outcomes | Literature review | Detecting risk, assessing risk and decision-making |
| [128]* | Morriss | 2013 | USA | To estimate the contribution of insurance status to access to health care resources and to neonatal survival outcome | Retrospective study using a database | Aligning risk and resources |
| [129] | Morriss | 2018 | USA | To estimate the annual rate of inter-hospital transfers of pregnant and postpartum women and analyze associated patient and health system characteristics as measures of regionalized perinatal care performance | Retrospective study reviewing case records | Detecting risk, assessing risk and decision-making |
| [130]* | Nuovo | 1985 | USA | A risk-scoring system was evaluated to determine whether a request for obstetric or pediatric assistance could be predicted | Survey | Detecting risk, assessing risk and decision-making |
| [131]* | Offerhaus | 2015a | Netherlands | To study whether an increase in intrapartum referrals in primary midwife-led care births in the Netherlands is accompanied by an increase in caesarean sections | Retrospective study using a database | Ensuring safety |
| [132]* | Offerhaus | 2015b | Netherlands | To explore the influence of risk perception, policy on routine labor management, and other midwife related factors on intrapartum referral decisions of midwives | Questionnaire | Detecting risk, assessing risk and decision-making |
| [133]* | Offerhaus | 2015c | Netherlands | To describe the variation in intrapartum referral rates in midwifery practices in and to explore the association between the practice referral rate and a woman's chance of an instrumental birth | Retrospective study using a database | Ensuring safety |
| [134]* | Papiernik | 1995 | France | To evaluate regionalized perinatal care programs in countries where they exist | Literature review | Aligning risk and resources |
| [135] | Patterson | 2017 | New Zealand | To explore retrospectively the choice of birth place decisions and the labor and birth experiences of a sample of women resident in remotely zoned, rural areas | Interviews | Detecting risk, assessing risk and decision-making |
| [136]* | Peddle | 1983 | Canada | Reviews the development of a regionalization programme and demonstrated by consumer utilization and perinatal mortality trends throughout the geographic area served | Retrospective cohort study using a database | Aligning risk and resources |
| [137]* | Perdok | 2015 | Netherlands | To examine reasons for referral, management of labor, and maternal and neonatal outcomes among women who were referred during labor | Retrospective cohort study using a database | Ensuring safety |
| [138] | Perdok | 2016 | Netherlands | To gain insight into the level of consensus among maternity care professionals about facilitators and barriers related to integration of midwife-led and obstetrician-led care | Questionnaire | Detecting risk, assessing risk and decision-making |
| [139] | Persson | 2004 | Greenland | To evaluate reform of health services | Retrospective study reviewing case records | Ensuring safety |
| [140] | Phillippi | 2019 | USA | To describe and disseminate the planning checklist to assist other health systems that provide team-based care with midwife and physician maternity care providers | Survey | Detecting risk, assessing risk and decision-making |
| [141] | Posthumus | 2016a | Netherlands | To explore the dynamics of obstetric  care utilization we investigated the joint association of hospital density and individual characteristics with prototype obstetric interventions | Retrospective cohort study using a database | Detecting risk, assessing risk and decision-making |
| [142] | Posthumus | 2016b | Netherlands | To investigate the associations between the use of a scorecard and risk factors and preterm birth, small for gestational age and a low Apgar score | Prospective cohort study using case records | Detecting risk, assessing risk and decision-making |
| [143] | Prentice | 1989 | England | To audit the outcome of pregnancies booked for confinement in a general practitioner maternity unit in a district general hospital | Retrospective review of case records | Ensuring safety |
| [144] | Quinn | 2014 | Australia | To better understand the local context and progress in delivering recommendations to improve  maternity services for women in remote communities | Questionnaire | Detecting risk, assessing risk and decision-making |
| [145] | Radomsky | 1995 | Canada | To review obstetric care provided by family physicians and to determine why they transfer patients to obstetricians | Retrospective review of case | Detecting risk, assessing risk and decision-making |
| [146]* | Ravelli | 2008 | Netherlands | To gain insight in recent perinatal mortality and their relation with important risk factors, risk groups  and risk selection among pregnant women | Retrospective cohort study using a database | Ensuring safety |
| [147]* | Reddy | 2004 | England | To evaluate the safety of this birth setting for low-risk deliveries based on our hospital protocol | Retrospective review of case records | Ensuring safety |
| [148] | Reilly | 2018 | Australia | To report the clinical features and outcomes of women referred to coordinated in-utero service with a primary diagnosis of pre-eclampsia, and subsequently transferred in-utero | Retrospective review of case records | Ensuring safety |
| [149] | Reither | 2018 | USA | To investigate midwifery management of pregnant women with obesity | Survey | Detecting risk, assessing risk and decision-making |
| [150]* | Ressl | 2015 | Canada | To evaluate the effectiveness of the current detection process of breech | Retrospective review of case records and a prospective survey | Detecting risk, assessing risk and decision-making |
| [151]* | Reynolds | 1988 | England | To examine the effectiveness of antenatal risk prediction based on maternal characteristics | Retrospective cohort study using a database | Detecting risk, assessing risk and decision-making |
| [152]* | Richardson | 1984 | USA | To study decisions to refer obstetric patients | Survey | Detecting risk, assessing risk and decision-making |
| [153]* | Richardson | 1985 | USA | To describe issues in modern perinatal care in an effort to explore larger points concerning the efficiency and rationality of the way services are organized and rendered in today's evolving health care system | Literature review | Ensuring safety |
| [154]* | Rijnders | 2008 | Netherlands | To investigate women’s views of their birth experience three years after the event | Questionnaire | Ensuring safety |
| [155]* | Roberts | 2000 | Australia | To quantify and describe antenatal transfers of rural women to perinatal centers, and among transferred women, to assess the use of selected evidence-based therapies and explore the potential predictors of preterm and imminent birth | Retrospective cohort study using a database | Aligning risk and resources |
| [156] | Rogers | 2010 | England | To determine outcomes for women booked at a stand-alone birth center | Prospective cohort study | Ensuring safety |
| [157] | Romijn | 2016 | Netherlands | To gain insight into similarities and differences between midwives and obstetricians in the assessment of a prolonged first stage of labor and the decision to refer a woman to a clinical setting | Survey | Detecting risk, assessing risk and decision-making |
| [158]* | Rooks | 1989 | USA | To study the safety of birth centers | Survey | Ensuring safety |
| [159] | Rooks | 1992a | USA | To examine the effectiveness of the birth centers’ prenatal screening process and to predict the impact of considering certain categories of women ineligible for birth center care | Survey | Ensuring safety |
| [160] | Rooks | 1992b | USA | to determine whether it would be possible to reduce the incidence of serious complications in birth centers by referring all women with certain prenatal complications to other sources of intrapartum care | Survey | Ensuring safety |
| [161]* | Rosenblatt | 1988 | USA | To evaluated the extent to which the regionalization of perinatal care has succeeded in concentrating high-risk pregnancies in technologically appropriate referral centers and in reducing differences in neonatal outcome among hospitals |  | Aligning risk and resources |
| [162]* | Rowe | 2012 | England | To examine the proportion of women transferred from midwifery units, the reasons for transfer and the sociodemographic and clinical factors associated with transfer | Prospective cohort study | Ensuring safety |
| [163] | Rowe | 2016 | England | To compare vaginal birth rates in women planning vaginal birth after caesarean at home versus in an obstetric unit and explore transfer rates in women planning home vaginal birth after caesarean | Prospective cohort study | Ensuring safety |
| [164] | Ryan | 1989 | England | To determine extent of maternal morbidity associated with in utero transfer | Retrospective review of case records | Ensuring safety |
| [165] | Scherjon | 1986 | Netherlands and Denmark | To compare two types of obstetric organizations | Retrospective cohort study using two databases | Aligning risk and resources |
| [166] | Scherman | 2008 | Australia | To describe a midwifery-led model of care in and the outcomes | Prospective study | Ensuring safety |
| [167] | Schmidt | 2002 | Norway | To report the short-term outcome for the mothers and newborns for all pregnancies accepted for birth at maternity homes | Prospective study | Ensuring safety |
| [168]* | Schuit | 2015 | Netherlands | To identify risk indicators for referral during labor from community midwife to a gynecologist | Prospective cohort study using a database | Detecting risk, assessing risk and decision-making |
| [169]* | Schwartz | 2000 | USA | To assess use of high technology care among women with high-risk pregnancies | Survey | Aligning risk and resources |
| [170] | Scupholme | 1986 | USA | To evaluate the relative safety of a birth center | Prospective matched paired study | Ensuring safety |
| [171] | Shaw | 2005 | UK (England, Ireland, Scotland, Wales) | To document the problems that callers to a helpline report having when trying to arrange home births and to explore the strategies the call-taker uses in helping women to exercise their right to birth at home | Audio-recordings | Detecting risk, assessing risk and decision-making |
| [172]* | Shenai | 1991 | USA | To test the hypothesized that successful implementation of regionalized perinatal care results in early identification and antenatal referral of high-risk neonates, and also improved stabilization before and during transport of those transferred postnatally | Retrospective study | Aligning risk and resources |
| [173]* | Sidhu | 1989 | Ireland | To compare the mortality in babies  refused admission to a regional perinatal center with that in babies accepted for intensive care in the center | Retrospective study using case records | Aligning risk and resources |
| [174]* | Sloan | 2008 | Canada | To compare the characteristics of a consultation-liaison services with similar services in different parts of the world | Retrospective study of case records | Ensuring safety |
| [175]* | Smit | 1997 | Netherlands | To compare referrals and reasons for referral during pregnancy and labor, mode of delivery and obstetric outcome of first births in women 35 years and older with women 20-30 years old | Explorative prospective cohort study | Detecting risk, assessing risk and decision-making |
| [176]* | Smit | 1998 | Netherlands | To compare referrals and reasons for referral during pregnancy and labor, mode of delivery and obstetric outcome of first births in women 35 years and older with women 20᎐30 years old | Prospective cohort study | Detecting risk, assessing risk and decision-making |
| [177]* | Smit | 2014 | Netherlands | To study the use of a pulse oximetry for midwives to assess neonatal condition after birth | Prospective observational study | Detecting risk, assessing risk and decision-making |
| [178] | Snowden | 2016 | USA | To assess maternal outcomes and fetal and neonatal outcomes according to the planned place of delivery | Population based retrospective cohort study using a database | Ensuring safety |
| [179]* | Stern | 1992 | Australia | To examine the outcomes of pregnancy and labor in a group of women who requested alternative birthing care and who were identified antenatally as being a ‘low-risk’ population | Retrospective study using case records | Ensuring safety |
| [180] | Stewart | 2017 | Australia | To present barriers to optimal in utero transfer of the high-risk fetus to a tertiary perinatal center and to discuss the principles to address these barriers | Literature review and authors’ experiences | Detecting risk, assessing risk and decision-making |
| [181]* | Stolp | 2015 | Netherlands | To evaluate the entire process of ambulance referral, from dispatcher call to hospital arrival | Prospective cohort study using case records and medical charts | Aligning risk and resources |
| [182] | Street | 1991 | England | To assess the effects of a revised obstetric booking policy whereby all low risk pregnant women received their antenatal care entirely in the community | Retrospective review of case records | Ensuring safety |
| [183] | Strobino | 2003 | USA | To describe perinatal linkages among hospitals, changes in their numbers and their impact on relationships among high-risk providers in local communities | Interviews | Aligning risk and resources |
| [184]* | Styles | 2011 | Scotland | To examine whether midwives' decision making during the intrapartum period was affected by midwives' attitudes towards risk; specifically, whether those midwives scoring highly on risk propensity would delay referring/transferring a woman in labor, compared with those who have a lower propensity for risk | Vignettes and a questionnaire | Detecting risk, assessing risk and decision-making |
| [185]* | Sullivan | 1995 | USA | To describe models for collaborative care for home birth, outlines the process of developing a collaborative practice agreement and discusses specific factors to consider when forming a relationship | Literature review | Detecting risk, assessing risk and decision-making |
| [186]* | Suzuki | 2009 | Japan | To examined obstetric outcomes of women with “low-risk” pregnancies related to the referral from midwives to obstetricians a hospital | Retrospective cohort study using case record | Ensuring safety |
| [187]* | Suzuki | 2014 | Japan | To perform trend analysis of primary midwife-led delivery care for ‘low risk’ pregnant women at our hospital | Retrospective cohort study using case record | Ensuring safety |
| [188] | Symon | 2010 | UK (England, Ireland, Scotland, Wales) | To examine independent midwives’ management and decision making in instances of perinatal death that occurred at term | Mixed methods consisting of a retrospective cohort study using case record and interviews | Detecting risk, assessing risk and decision-making |
| [189]* | Tilyard | 1988 | New Zealand | To assess the influence of the number of deliveries performed by each general practitioner obstetrician and the distance from specialist care | Retrospective review of case records | Detecting risk, assessing risk and decision-making |
| [190] | Tromp | 2009 | Netherlands | To study regional variation in perinatal mortality and to identify possible explanatory factors for the found differences | Retrospective cohort study using a database | Ensuring safety |
| [191]* | Tucker | 2003 | Scotland | To assess clinical appropriateness of model of care of small rural maternity units, including stand-alone midwife units | Retrospective review of case records | Aligning risk and resources |
| [192]* | Tucker | 2010 | Scotland | To assess the diagnosis and management of mild non-proteinuric hypertension in pregnancy in rural general practices against guideline recommendations | Survey and interviews | Detecting risk, assessing risk and decision-making |
| [193]* | Van Alten | 1989 | Netherlands | To investigate the procedures used for selecting maternity care and their results, studied a group of pregnant women who booked at a practice of freestanding midwives | Retrospective review of case records | Ensuring safety |
| [194] | Van der Kooy | 2016 | Netherlands | To address whether the introduction of a midwife-led birth center adjacent to the hospital combines the advantages of home and hospital deliveries, and whether the introduction of a midwife-led birth center leads to a different risk selection of women planning their delivery either at home, at the hospital or at the birth center | Retrospective review of case records | Aligning risk and resources |
| [195]* | Van Haaren | 2002 | Netherlands | To test the quality of obstetric care given by general practitioners | Retrospective review of case records | Ensuring safety |
| [196] | Van Otterloo | 2018 | USA | To improve the understanding of standardized and integrated system of risk-appropriate care | Literature review | Aligning risk and resources |
| [197]* | Van Stenus | 2017 | Netherlands | To explore questions around how clients experience transfers of care during pregnancy, childbirth, and the neonatal period, as well as how these experiences compare to the established quality of care aspects developed by the patient federation | Narratives | Ensuring safety |
| [198]* | Van Stenus | 2018 | Netherlands | To study if client experiences with perinatal healthcare differ between low-risk and high- risk women | Questionnaire | Ensuring safety |
| [199]* | Van Wagner | 2012 | Canada | To improve understanding of maternity services in remote communities | Retrospective review of case records | Ensuring safety |
| [200]* | Vause | 2014 | USA | To give and overview of risk scoring systems and lesion-specific indicators to identify cardiac disease | Literature review | Detecting risk, assessing risk and decision-making |
| [201]* | Vedam | 2007 | Canada | To provide a review of the aspects of intrapartum, postpartum, and neonatal midwifery management that can be provided similarly across birth sites, as well as those assessment, management, and therapeutic measures that need to be modified according to birth site | Literature review | Detecting risk, assessing risk and decision-making |
| [202] | Viisainen | 1994 | Finland | To study whether hospitals of different levels are equally safe places to give birth in a regionalized system of care | Cross sectional survey | Ensuring safety |
| [203]* | Vos | 2017 | Netherlands | To evaluate the implementation of a complex intervention in the antenatal healthcare field | Mixed methods consisting of observations and a survey | Detecting risk, assessing risk and decision-making |
| [204]* | Waldenström | 1997a | Sweden | To evaluate the effect of birth center care on women s health during pregnancy, birth, and 2 months postpartum by comparing the outcomes with those of women experiencing standard maternity care | Mixed methods consisting of a randomized controlled trial, review of case records and a questionnaire | Ensuring safety |
| [205] | Waldenström | 1997b | Sweden | To compare an in-hospital birth center with standard maternity care regarding medical interventions and maternal and infant outcome | Mixed methods consisting of a randomized controlled trial, review of case records and a questionnaire | Ensuring safety |
| [206]* | Wallace | 1995 | Scotland | To assess the quality and quantity of midwife-doctor referrals in an intrapartum setting | Retrospective review of case records and prospective observations | Detecting risk, assessing risk and decision-making |
| [207]* | Wiegers | 1998 | Netherlands | To examine the differences between women choosing to give birth at home and those choosing a hospital birth to assess their experiences and the extent to which they were influenced by referral (and transfer) to specialist care | Questionnaire | Detecting risk, assessing risk and decision-making |
| [208]* | Woodcock | 1990 | Australia | To obtain accurate and complete data on homebirths to assist the formulation of appropriate homebirth policies | Retrospective review of case records | Ensuring safety |
| [209]* | Woodhart | 2018 | Australia | To explore the experiences of a small cohort of women during and following their transfer for higher level pregnancy and neonatal care | Interviews | Ensuring safety |
| [210]* | Wright | 2013 | USA | To determine their knowledge, patterns of care and treatment preferences for women with placenta accreta | Cross-sectional survey | Detecting risk, assessing risk and decision-making |

* First 125 papers of which data was extracted

1. Aalfs CM, Smets EMA, de Haes HCJM, Leschot NJ. Referral for genetic counselling during pregnancy: limited alertness and awareness about genetic risk factors among GPs. Fam Pract. 2003;20(2):135–41.

2. Accortt EE, Wong MS. It Is Time for Routine Screening for Perinatal Mood and Anxiety Disorders in Obstetrics and Gynecology Settings. Obstet Gynecol Surv. 2017;72(9):553–68.

3. Allen C, Greene R, Higgins J. Audit of antenatal clinic for high-risk obstetric patients; activity and outcomes. Ir Med J. 2007;100(9):591–3.

4. Amelink-Verburg MP, Verloove-Vanhorick SP, Hakkenberg RMA, Veldhuijzen IME, Bennebroek Gravenhorst J, Buitendijk SE. Evaluation of 280 000 cases in Dutch midwifery practices: a descriptive study. BJOG. 2008;115:570–8.

5. Amelink-Verburg MP, Rijnders MEB, Buitendijk SE. A trend analysis in referrals during pregnancy and labour in Dutch midwifery care 1988-2004. BJOG. 2009;116:923–32.

6. Amelink-Verburg MP, Buitendijk SE. Pregnancy and labour in the Dutch maternity care system: what is normal? The role division between midwives and obstetricians. J Midwifery Womens Health. 2010;55(3):216–25.

7. Ammari F, Gregory R. Screening for gestational diabetes in a population at high risk. Pract Diabetes Int. 1995;13(5):150–2.

8. Anderson RE, Murphy PA. Outcomes of 11,788 planned home births attended by certified nurse-midwives. A retrospective descriptive study. J Nurse Midwifery. 1995;40(6):483–92.

9. Badgery-Parker T, Ford JB, Jenkins MG, Morris JM, Roberts CL. Patterns and outcomes of preterm hospital admissions during pregnancy in NSW, 2001–2008. Med J Aust. 2012;196(4):261–5.

10. Bahry VJ, Fullerton JT, Lops VR. Provision of comprehensive perinatal services through rural outreach: a model program. J Rural Heal. 1989;5(4):387–96.

11. Baird AG, Jewell D, Walker J. Management of labour in an isolated rural maternity hospital. BMJ. 1996;312:223–6.

12. Bais JMJ, Eskes M, Pel M, Bonsel GJ, Bleker OP. Effectiveness of detection of intrauterine growth retardation by abdominal palpation as screening test in a low risk population: an observational study. Eur J Obstet Gynecol Reprod Biol. 2004;116:164–9.

13. Baker SL, Kronenfeld JJ. High risk channeling to improve medicaid maternal and infant care. J Health Soc Policy. 1992;3(4):29–49.

14. Baldwin L-M, Hart G, Lloyd M, Fordyce M, Rosenblatt RA. Defensive medicine and obstetrics. JAMA J Am Med Assoc. 1995 Nov;274(20):1606–10.

15. Baldwin A, Harvey C, Willis E, Ferguson B, Capper T. Transitioning across professional boundaries in midwifery models of care: a literature review. Women and Birth. 2019;32:195–203.

16. Bernitz S, Rolland R, Blix E, Jacobsen M, Sjøborg K, Øian P. Is the operative delivery rate in low-risk women dependent on the level of birth care? A randomised controlled trial. BJOG. 2011;118:1357–64.

17. Bernstein JA, McCloskey L, Gebel CM, Iverson RE, Lee-Parritz A. Lost opportunities to prevent early onset type 2 diabetes mellitus after a pregnancy complicated by gestational diabetes. BMJ Open Diabetes Res Care. 2016 Jun 17;4:e000250.

18. Binder S, Hill K, Meinzen-Derr J, Greenberg JM, Narendran V. Increasing VLBW deliveries at subspecialty perinatal centers via perinatal putreach. Pediatrics. 2011;127(3):487–93.

19. Biro M, Lumley J. The safety of team midwifery: the first decade of the Monash Birth Centre. Med J Aust. 1991;155:478–80.

20. Blix E, Kumle MH, Ingversen K, Huitfeldt AS, Hegaard HK, Ólafsdóttir ÓÁ, Øian P, Lindgren H. Transfers to hospital in planned home birth in four Nordic countries - a prospective cohort study. Acta Obstet Gynecol Scand. 2016;95:420–8.

21. Blondel B, Papiernik E, Delmas D, Künzel W, Weber T, Maier R, Kollée L, Zeitlin J. Organisation of obstetric services for very preterm births in Europe: results from the MOSAIC project. BJOG. 2009 Sep;116(10):1364–72.

22. Bode MM, O’Shea MT, Metzguer KR, Stiles AD. Perinatal regionalization and neonatal mortality in North Carolina, 1968-1994. Am J Obstet Gynecol. 2001;184(6):1302–7.

23. Bodner-Adler B, Kimberger O, Griebaum J, Husslein P, Bodner K. A ten-year study of midwife-led care at an Austrian tertiary care center: a retrospective analysis with special consideration of perineal trauma. BMC Pregnancy Childbirth. 2017;17:357.

24. Bolbocean C, Wintermark P, Shevell MI, Oskoui M. Perinatal regionalization and implications for long-term health outcomes in cerebral palsy. Can J Neurol Sci / J Can des Sci Neurol. 2016;1–6.

25. Bovbjerg ML, Cheyney M, Brown J, Cox KJ, Leeman L. Perspectives on risk: Assessment of risk profiles and outcomes among women planning community birth in the United States. Birth. 2017;44:209–21.

26. Britt DW, Eden RD, Evans MI. Matching risk and resources in high-risk pregnancies. J Matern Neonatal Med. 2006;19(10):645–50.

27. Bronstein JM, Ounpraseuth S, Jonkman J, Lowery CL, Fletcher D, Nugent RR, Hall RW. Improving Perinatal Regionalization for Preterm Deliveries in a Medicaid Covered Population: Initial Impact of the Arkansas ANGELS Intervention. Health Serv Res. 2011;46(4):1082–103.

28. Butler MM, Sheehy L, Kington MM, Walsh MC, Brosnan MC, Murphy M, Naughton C, Drennan J, Barry T. Evaluating midwife-led antenatal care: Choice, experience, effectiveness, and preparation for pregnancy. Midwifery. 2015;31:418–25.

29. Carolan M, Hodnett E. Discovery of soft markers on fetal ultrasound: maternal implications. Midwifery. 2009;25:654–64.

30. Chambliss LR, Daly C, Medearis AL, Ames M, Kayne M, Paul R. The role of selection bias in comparing cesarean birth rates between physician and midwifery management. Obstet Gynecol. 1992;80(2):161–5.

31. Cohen L, Schaeffer M, Davideau J-L, Tenenbaum H, Huck O. Obstetric knowledge, attitude, and behavior concerning periodontal diseases and treatment needs in pregnancy: influencing factors in France. J Periodontol. 2015;86(3):398–405.

32. Cooke HM, Waters DL, Dyer K, Lawler J, Picone D. Development of a best practice model of midwifery-led antenatal care. Aust Midwifery. 2004;17(2):21–5.

33. Cordero L, Schurman S, Zuspan FP. Appropriateness of antenatal referrals to a regional perinatal center. J Perinatol. 1989;9(1):38–42.

34. Craig AS, Berg AO, Kirkwood CR. Obstetrie consultations during labor and delivery in a university based family practice. J Fam Pract. 1985;20(5):481–5.

35. Crotty M, Ramsay AT, Smart R, Chan A. Planned homebirths in South Australia 1976-1987. Med J Aust. 1990;153:664–71.

36. Daemers DOA, Wijnen HAA, van Limbeek EBM, Budé LM, Nieuwenhuijze MJ, Spaanderman MEA, De Vries RG. The impact of obesity on outcomes of midwife-led pregnancy and childbirth in a primary care population: a prospective cohort study. BJOG. 2014;121:1403–14.

37. David M, Berg G, Werth I, Pachaly J, Mansfeld A, Kentenich H. Intrapartum transfer from a birth centre to a hospital – reasons, procedures, and consequences. Acta Obstet Gynecol Scand. 2006;85:422–8.

38. de Galan-Roosen AEM, Kuijpers JC, Mackenbach JP. Perinatal mortality in Delft and environs, 1983 - 1992: further decrease possible by specific attention to lethal congenital anomalies and placental insufficiency. Ned Tijdschr voor Geneeskd. 1999;143:152–7.

39. de Jonge A, van der Goes BY, Ravelli ACJ, Amelink-Verburg MP, Mol BW, Nijhuis JG, Gravenhorst JB, Buitendijk SE. Perinatal mortality and morbidity in a nationwide cohort of 529 688 low-risk planned home and hospital births. BJOG. 2009;116:1177–84.

40. de Jonge A, Rijnders M, Agyemang C, van der Stouwe R, den Otter J, Van den Muijsenbergh METC, Buitendijk S. Limited midwifery care for undocumented women in the Netherlands. J Psychosom Obstet Gynecol. 2011;32(4):182–8.

41. de Jonge A, Mesman JAJM, Mannien J, Zwart JJ, van Dillen J, van Roosmalen J. Severe adverse maternal outcomes among low risk women with planned home versus hospital births in the Netherlands: nationwide cohort study. BMJ. 2013;346:f3263.

42. de Jonge A, Mesman JAJM, Manniën J, Zwart JJ, Buitendijk SE, van Roosmalen J, van Dillen J. Severe adverse maternal outcomes among women in midwife-led versus obstetrician-led care at the onset of labour in the Netherlands: a nationwide cohort study. PLoS One. 2015;10(5):e0126266.

43. De Reu PAOM, Oosterbaan HP, Smits LJM, Nijhuis JG. Avoidable mortality in small-for-gestational-age children in the Netherlands. J Perinat Med. 2010;38(3):311–8.

44. de Weerd S, Wouters MGAJ, Mom-Boertjens J, Bos KL. Preconception advice: evaluation of an outpatients’ clinic at a university hospital. Nederlands Tijdschrift voor de Geneeskunde. 2001.

45. DeJong RN, Carr KC. An out-of-hospital birth center using university referral. Obstet Gynecol. 1981 Dec;58(6):703–7.

46. Delaney-Black V, Lubchenco LO, Joseph Butterfield L, Goldson E, Koops BL, Lazotte DC. Outcome of very-low-birth-weight infants: are populations of neonates inherently different after antenatal versus neonatal referral? Am J Obstet Gynecol. 1989;160(3):545–52.

47. Dencker A, Smith V, McCann C, Begley C. Midwife-led maternity care in Ireland – a retrospective cohort study. BMC Pregnancy Childbirth. 2017;17:101.

48. Dijkstra K, Kuyvenhoven M, Verheij T, Iedema H, Springer M, Visser G. Dreigende vroeggeboorte; opvattingen en werkwijze van verloskundigen, huisartsen en gynaecologen [Threatened pre-term delivery: opinions and working methods of midwives, GPs and gynaecologists]. Huisarts Wet. 2003;46(3):129–33.

49. Donohue PK, Boss RD, Shepard J, Graham E, Allen MC. Intervention at the border of viability – perspective over a decade. Arch Pediatr Adolesc Med. 2009 Oct;163(10):902–6.

50. Eden RD, Eden RD, Penka A, Britt DW, Landsberger EJ, Evans MI. Re-evaluating the role of the MFM specialist: lead, follow, or get out of the way. J Matern Neonatal Med. 2005;18(4):253–8.

51. Eide BI, Nilsen ABV, Rasmussen S. Births in two different delivery units in the same clinic – a prospective study of healthy primiparous women. BMC Pregnancy Childbirth. 2009;9:25.

52. Engjom HM, Morken N-H, Høydahl E, Norheim OF, Klungsøyr K. Risk of eclampsia or HELLP-syndrome by institution availability and place of delivery – a population-based cohort study. Pregnancy Hypertens. 2018;14:1–8.

53. Eskes M, Waelput AJM, Scherjon SA, Bergman KA, Abu-Hanna A, Ravelli ACJ. Small for gestational age and perinatal mortality at term: An audit in a Dutch national cohort study. Eur J Obstet Gynecol Reprod Biol. 2017;215:62–7.

54. Evers ACC, Brouwers HAA, Hukkelhoven CWPM, Nikkels PGJ, Boon J, van Egmond-Linden A, Hillegersberg J, Snuif YS, Sterken-Hooisma S, Bruinse HW, Kwee A. Perinatal mortality and severe morbidity in low and high risk term pregnancies in the Netherlands: prospective cohort study. BMJ. 2010;341:c5639.

55. Evers ACC, Brouwers HAA, Nikkels PGJ, Boon J, van Egmond-Linden A, Groenendaal F, Hart C, Hillegersberg J, Snuif YS, Sterken-Hooisma S, Steins Bisschop CN, Westerhuis MEMH, Bruinse HW, Kwee A. Substandard care in delivery-related asphyxia among term infants: prospective cohort study. Acta Obstet Gynecol Scand. 2013;92:85–93.

56. Ferndale D, Meuter RFI, Watson B, Gallois C. ‘You don’t know what’s going on in there’: a discursive analysis of midwifery hospital consultations. Health Risk Soc. 2017;19(7–8):411–31.

57. Ferrazzi E, Visconti E, Paganelli AM, Campi CM, Lazzeri C, Cirillo F, Livio S, Piola C. The outcome of midwife-led labor in low-risk women within an obstetric referral unit. J Matern Neonatal Med. 2015;28(13):1530–6.

58. Finnström O, Berg G, Norman A, Olausson PO. Size of delivery unit and neonatal outcome in Sweden. A catchment area analysis. Acta Obstet Gynecol Scand. 2006;85:63–7.

59. Fleissig A, Kroll D, McCarthy M. Is community-led maternity care a feasible option for women assessed at low risk and those with complicated pregnancies? Results of a population based study in South Camden, London. Midwifery. 1996;12:191–7.

60. Ford C, Iliffe S, Franklin O. Outcome of planned home births in an inner city practice. BMJ. 1991 Dec 14;303:1517–9.

61. Fullerton JT, Jackson D, Snell BJ, Besser M, Dickinson C, Garite T. Transfer rates from freestanding birth centers – a comparison with the National Birth Center Study. J Nurse Midwifery. 1997;42(1):9–16.

62. Garite TJ, Snell BJ, Walker DL, Darrow VC. Development and experience of a university based, freestanding birthing center. Obstet Gynecol. 1995 Sep;86(3):411–6.

63. Gaudineau A, Sauleau E-A, Nisand I, Langer B. Obstetric and neonatal outcomes in a home-like birth centre: a case – control study. Arch Gynecol Obstet. 2013;287:211–6.

64. Geerts Caroline C, Trudy K, Lagro-Janssen Antoine LM, Twisk Jos WR, Dillen V, Jeroen, Jonge D, Ank. Birth setting, transfer and maternal sense of control: results from the DELIVER study. BMC Pregnancy Childbirth. 2014;14:27.

65. George A, Dahlen HG, Blinkhorn A, Ajwani S, Bhole S, Ellis S, Yeo A, Elcombe E, Johnson M. Evaluation of a midwifery initiated oral health-dental service program to improve oral health and birth outcomes for pregnant women: a multi-centre randomised controlled trial. Int J Nurs Stud. 2018;82:49–57.

66. Giles W, Bisits A, Knox M, Madsen G, Smith R. The effect of fetal fibronectin testing on admissions to a tertiary maternal-fetal medicine unit and cost savings. Am J Obstet Gynecol. 2000;182:439–42.

67. Gillespie M, Sinclair M, Stockdale J, Bunting B, Condell J. Online educational resources for health professionals caring for pregnant women with heart disease: a scoping literature review using Arksey and O’Malley’s methodological framework. Evid Based Midwifery. 2018;16(2):55–61.

68. Godbole G, Irish D, Basarab M, Mahungu T, Fox-Lewis A, Thorne C, Jacobs M, Dusheiko G, Rosenberg WMC, Suri D, Millar AD, Nastouli E. Management of hepatitis B in pregnant women and infants: a multicentre audit from four London hospitals. BMC Pregnancy Childbirth. 2013;13:222.

69. Goh A, Browning Carmo K, Morris J, Berry A, Wall M, Abdel-Latif M. Outcomes of high-risk obstetric transfers in New South Wales and the Australian capital territory: the high-risk obstetric transfer study. Aust New Zeal J Obstet Gynaecol. 2015;1–6.

70. Govaerts L, Srebniak M, Diderich K, Joosten M, Riedijk S, Knapen M, Go A, Papatsonis D, de Graaf K, Toolenaar T, van der Steen S, Huijbregts G, Knijnenburg J, de Vries F, Van Opstal D, Galjaard R-J. Prenatal diagnosis of susceptibility loci for neurodevelopmental disorders - genetic counseling and pregnancy outcome in 57 cases. Prenat Diagn. 2017;37:73–80.

71. Grigg CP, Tracy SK, Tracy M, Schmied V, Monk A. Transfer from primary maternity unit to tertiary hospital in New Zealand – timing, frequency, reasons, urgency and outcomes: part of the Evaluating Maternity Units study. Midwifery. 2015;31:879–87.

72. Gyte G, Dodwell M, Newburn M, Sandall J, Macfarlane A, Bewley S. Estimating intrapartum-related perinatal mortality rates for booked home births: when the ‘best’ available data are not good enough. BJOG. 2009;116:933–42.

73. Hein HA, Burmeister LF. The effect of ten years of regionalized perinatal health care in Iowa, U.S.A. Eur J Obstet Gynecol Reprod Biol. 1986;21:33–48.

74. Hemminki E, Malin M, Kojo-Austin H. Prenatal care in Finland: from primary to tertiary health care? Int J Heal Serv. 1990;20(2):221–32.

75. Hemminki E, Heino A, Gissler M. Should births be centralised in higher level hospitals? Experiences from regionalised health care in Finland. BJOG. 2011;118:1186–95.

76. Hollingworth J, Pietsch R, Epee-Bekima M, Nathan E. Time to delivery: transfers for threatened preterm labour and prelabour rupture of membranes in Western Australia. Aust J Rural Health. 2018;26:42–7.

77. Holt J, Vold IN, Backe B, Johansen MV, Øian P. Child births in a modified midwife managed unit: Selection and transfer according to intended place of delivery. Acta Obstet Gynecol Scand. 2001;80:206–12.

78. Hopkins MK, Goldstein SA, Ward CC, Kuller JA. Evaluation and management of aternal congenital ceart disease: a review. Obstet Gynecol Surv. 2018;73(2):116–24.

79. Hueston WJ, The Factors Meeting Cesarean Section (FACS) Study Group. Obstetric referral in family practice. J Fam Pract. 1994 Apr;38(4):368–72.

80. Humphrey MD, Foxcroft KF, Callaway LK. Obstetric risk score – revalidated for triaging high-risk pregnancies in rural areas. Aust New Zeal J Obstet Gynaecol. 2017;57:63–7.

81. Hundley VA, Cruickshank FM, Lang GD, Glazener CMA, Milne JM, Turner M, Blyth D, Mollison J, Donaldson C. Midwife managed delivery unit: a randomised controlled comparison with consultant led care. BMJ. 1994 Nov 26;309(6966):1400–4.

82. Hutchinson FH, Davies MW. Time-to-delivery after maternal transfer to a tertiary perinatal centre. Biomed Res Int. 2014;325919.

83. Hutton EK, Reitsma AH, Kaufman K. Outcomes associated with planned home and planned hospital births in low-risk women attended by midwives in ontario, Canada, 2003-2006: a retrospective cohort study. Birth. 2009;36(3):180–9.

84. Jackson CJ, Bosio P, Habiba M, Waugh J, Kamal P, Dixon-Woods M. Referral and attendance at a specialist antenatal clinic: qualitative study of women’s views. BJOG. 2006;113:909–13.

85. James A, Endacott R, Stenhouse E. Maternity High Dependency Care (MHDC) in Obstetric Units remote from tertiary referral centres; findings of a modified Delphi study. Evid Based Midwifery. 2017;15(4):120–7.

86. Janssen PA, Lee SK, Ryan ER, Saxell L. An evaluation of process and protocols for planned home birth attended by regulated midwives in British Columbia. J Midwifery Womens Health. 2003;48(2):138–45.

87. Jeffery J, Hewison A, Goodwin L, Kenyon S. Midwives’ experiences of performing maternal observations and escalating concerns: a focus group study. BMC Pregnancy Childbirth. 2017;17:282.

88. Jordan JM, Gaspar D. Family practice obstetrics in a teaching hospital – Does a tertiary care environment make a dfference? Can Fam Physician. 1995;41(April):610–5.

89. Kim JJ, La Porte LM, Corcoran M, Magasi S, Batza J, Silver RK. Barriers to mental health treatment among obstetric patients at risk for depression. Am J Obstet Gynecol. 2010;202:312.e1-312.e5.

90. Kirke AB. How safe is GP obstetrics? An assessment of antenatal risk factors and perinatal outcomes in one rural practice. Rural Remote Health. 2010;10:1545.

91. Knight M. The findings of the MBRRACE-UK confidential enquiry into maternal deaths and morbidity. Obstet Gynaecol Reprod Med. 2018;29(1):21–3.

92. Knox GE, Schnitker KA. In-utero transport. Clin Obstet Gynecol. 1984;27(1):11–6.

93. Kollée LAA, Den Ouden AL, Drewes JG, Brouwers HAA, Verwey RA, Verloove-Vanhorick SP. Toename van perinatale verwijzing naar regionale centra bij vroeggeboorte in Nederland: vergelijking van 1983 en 1993 [Increased perinatal referral to regional centres of premature infants in the Netherlands: comparison of 1983 and 1993]. Ned Tijdschr voor Geneeskd. 1998;142(3):131–4.

94. Koshida S, Ono T, Tsuji S, Murakami T, Takahashi K. Recommendations for preventing stillbirth: a regional population-based study in Japan during 2007-2011. Tohoku J Exp Med. 2015;235:145–9.

95. Kruske S, Schultz T, Eales S, Kildea S. A retrospective, descriptive study of maternal and neonatal transfers, and clinical outcomes of a primary maternity unit in rural Queensland, 2009–2011. Women and Birth. 2015;28:30–9.

96. Kruske S, Kildea S, Jenkinson B, Pilcher J, Robin S, Rolfe M, Kornelsen J, Barclay L. Primary maternity units in rural and remote Australia: results of a national survey. Midwifery. 2016;40:1–9.

97. Kuliukas L, Hauck Y, Duggan R, Lewis L. The phenomenon of intrapartum transfer from a western Australian birth centre to a tertiary maternity hospital: the overall experiences of partners. Midwifery. 2015;31:e87–93.

98. Kwong AJ, Chang MS, Tuomala RE, Riley LE, Robinson JN, Mutinga ML, Andersson KL, Brown Jr. RS, Oken E, Ukomadu C, Rutherford AE. Peripartum care for mothers diagnosed with hepatitis B during pregnancy: a survey of provider practices. Matern Child Health J. 2018;22:1345–51.

99. Lagendijk J, Vos AA, Bertens LCM, Denktas S, Bonsel GJ, Steyerberg EW, Been J V., Steegers EAP. Antenatal non-medical risk assessment and care pathways to improve pregnancy outcomes: a cluster randomised controlled trial. Eur J Epidemiol. 2018;33:579–89.

100. Lalor JG, Devane D, Begley CM. Unexpected diagnosis of fetal abnormality: women’s encounters with caregivers. Birth. 2007;34(1):80–8.

101. Laube DW. Experience with an alternative birth center in a university hospital. J Reprod Med. 1983;391–6.

102. Lavender T, Alfirevic Z, Walkinshaw S. Effect of different partogram action lines on birth outcomes – a randomized controlled trial. Obstet Gynecol. 2006;108(2):295–302.

103. Law YYH, Lam K-Y. A randomized controlled Ttial comparing midwife-managed care and obstetrician-managed care for women assessed to be at low risk in the initial intrapartum period. J Obstet Gynaecol Res. 1999;25(2):107–12.

104. Leddy MA, Lawrence H, Schulkin J. Obstetrician-gynecologists and womens mental health: Findings of the collaborative ambulatory research network 2005-2009. Obstet Gynecol Surv. 2011;66(5):316–23.

105. Lennox CE. Transferring at-risk babies in-utero or neonatally: a decade’s experience from a peripheral consultant maternity unit. Health Bull (Raleigh). 1992;50(5):362–7.

106. Lessaris KJ, Annibale DJ, Southgate MW, Hulsey TC, Ohning BL. Effects of changing health care financial policy on very low birthweight neonatal outcomes. South Med J. 2002;95(4):426–30.

107. Lim JCES, Cauldwell M, Patel RR, Uebing A, Curry RA, Johnson MR, Gatzoulis MA, Swan L. Management of marfan syndrome during pregnancy: a real world experience from a joint cardiac obstetric service. Int J Cardiol. 2017;243:180–4.

108. Lubchenco LO, Joseph Butterfield L, Delaney-Black V, Goldson E, Koops BL, Lazotte DC. Outcome of very-low-birth-weight infants: does antepartum versus neonatal referral have a better impact on mortality, morbidity, or long-term outcome? Am J Obstet Gynecol. 1989;160(3):539–45.

109. Lundeen T. Intrapartum and Postpartum Transfers to a Tertiary Care Hospital from Out-of-Hospital Birth Settings: A Retrospective Case Series. J Midwifery Womens Health. 2016;61:242–8.

110. Maassen MS, Hendrix MJC, van Vugt HC, Veersma S, Smits F, Nijhuis JG. The choice of obstetric care by low-risk pregnant women in the Netherlands: implications for policy and management. Birth. 2008;35:277–82.

111. MacVicar J, Dobbie G, Owen-Johnstone L, Jgger C, Hopkins M, Kennedy J. Simulated home delivery in hospital: a randomised controlled trial. Br J Obstet Gynaecol. 1993;100:316–23.

112. Magann EF, Bronstein J, McKelvey SS, Wendel P, Smith DM, Lowery CL. Evolving trends in maternal fetal medicine referrals in a rural state using telemedicine. Arch Gynecol Obstet. 2012 Dec;286(6).

113. Mansbridge K. Nurse-to-nurse referral of patients in early pregnancy. Emerg Nurse. 2014;22(1):27–31.

114. Marlow N, Bennett C, Draper ES, Hennessy EM, Morgan AS, Costeloe KL. Perinatal outcomes for extremely preterm babies in relation to place of birth in England: the EPICure 2 study. Arch Dis Child - Fetal Neonatal Ed. 2014;99:F181–8.

115. Martijn L, Jacobs A, Amelink-Verburg M, Wentzel R, Buitendijk S, Wensing M. Adverse outcomes in maternity care for women with a low risk profile in The Netherlands: a case series analysis. BMC Pregnancy Childbirth. 2013;13:219.

116. Matthey S, Souter K, Mortimer K, Stephens C, Sheridan-Magro A. Routine antenatal maternal screening for current mental health: evaluation of a change in the use of the Edinburgh Depression Scale in clinical practice. Arch Womens Ment Health. 2016;19:367–72.

117. Mayer F, Bick D, Taylor C. Multidisciplinary care for pregnant women with cardiac disease: a mixed methods evaluation. Int J Nurs Stud. 2018;85:96–105.

118. McCool WF, Guidera M, Griffinger E, Sacan D. Closed claims analysis of medical malpractice lawsuits involving midwives: lessons learned regarding safe practices and the avoidance of litigation. J Midwifery Womens Health. 2015;60:437–44.

119. McIntyre MJ. Safety of non-medically led primary maternity care models: a critical review of the international literature. Aust Heal Rev. 2012;36(2):140–7.

120. McMurtrie J, Carling-Paul C, Teate A, Caplice S, Chapman M, Homer C. The St. George Homebirth Program: an evaluation of the first 100 booked women. Aust New Zeal J Obstet Gynaecol. 2009;49:631–6.

121. Mengel MB, Phillips WR. The quality of obstetric care in family practice: are family physicians as safe as obstetricians? J Fam Pract. 1987;24(2):159–64.

122. Meuli RL, Cohen LJ. Regionalization of perinatal care. West J Med. 1984;141(5):695–7.

123. Mito A, Arata N, Sakamoto N, Miyakoshi K, Waguri M, Osamura A, Kugishima Y, Metoki H, Yasuhi I. Present status of clinical care for postpartum patients with hypertensive disorders of pregnancy in Japan: findings from a nationwide questionnaire survey. Hypertens Pregnancy. 2015;34(2):209–20.

124. Monk A, Tracy M, Foureur M, Grigg C, Tracy S. Evaluating Midwifery Units (EMU): a prospective cohort study of freestanding midwifery units in New South Wales, Australia. BMJ Open. 2014;4:e006252.

125. Montgomery-Andersen RA, Willén H, Borup I. ‘There was no other way things could have been.’ Greenlandic women’s experiences of referral and transfer during pregnancy. Anthropol Med. 2010;17(3):301–13.

126. Morano S, Cerutti F, Mistrangelo E, Pastorino D, Benussi M, Costantini S, Ragni N. Outcomes of the first midwife-led birth centre in Italy: 5 years’ experience. Arch Gynecol Obstet. 2007;276:333–7.

127. Morley K. Epilepsy in pregnancy: the role of the midwife in risk management. Br J Midwifery. 2018 Sep;26(9):564–73.

128. Morriss FH. Increased risk of death among uninsured neonates. Health Serv Res. 2013;48(4):1232–55.

129. Morriss F. Interhospital transfers of maternal patients: cohort analysis of nationwide inpatient sample, 2011. Am J Perinatol. 2018;35:65–77.

130. Nuovo J. Clinical application of a high-risk scoring system on a family practice obstetric service. J Fam Pract. 1985;20(2):139–44.

131. Offerhaus PM, de Jonge A, van der Pal-de Bruin KM, Hukkelhoven CWPM, Scheepers PLH, Lagro-Janssen ALM. Change in primary midwife-led care in the Netherlands in 2000–2008: A descriptive study of caesarean sections and other interventions among 807,437 low risk births. Midwifery. 2015;31:648–54.

132. Offerhaus PM, Otten W, Boxem-Tiemessen JCG, de Jonge A, van der Pal-de Bruin KM, Scheepers PLH, Lagro-Janssen ALM. Variation in intrapartum referral rates in primary midwifery care in the Netherlands: a discrete choice experiment. Midwifery. 2015;31:e69–78.

133. Offerhaus PM, Geerts C, de Jonge A, Hukkelhoven CWPM, Twisk JWR, Lagro-Janssen ALM. Variation in referrals to secondary obstetrician-led care among primary midwifery care practices in the Netherlands: a nationwide cohort study. BMC Pregnancy Childbirth. 2015;15:42.

134. Papiernik E, Keith LG. The regionalization of perinatal care in France — description of a missing policy. Eur J Obstet Gynecol Reprod Biol. 1995;61:99–103.

135. Patterson J, Foureur M, Skinner J. Remote rural women’s choice of birthplace and transfer experiences in rural Otago and Southland New Zealand. Midwifery. 2017;52:49–56.

136. Peddle LJ, Brown H, Buckley J, Dixon W, Kaye J (MacDonald), Muise M, Rees E, Woodhams W, Young C. Voluntary regionalization and associated trends in perinatal care: The Nova Scotia Reproductive Care Program. Am J Obstet Gynecol. 1983;145(2):170–6.

137. Perdok H, Jans S, Verhoeven C, van Dillen J, Mol BW, de Jonge A. Intrapartum referral from primary to secondary care in the Netherlands: a retrospective cohort study on management of labor and outcomes. Birth. 2015;42(2):156–64.

138. Perdok H, Jans S, Verhoeven C, van Dillen J, Batenburg R, Mol BW, Schellevis F, de Jonge A. Opinions of professionals about integrating midwife- and obstetrician-led care in the Netherlands. Midwifery. 2016;37:9–18.

139. Persson G, Kern P. Reorganization of perinatal care in Greenland. Int J Circumpolar Health. 2004;63(sup2):397–8.

140. Phillippi JC, Holley SL, Thompson JL, Virostko K, Bennett K. A planning checklist for interprofessional consultations for women in midwifery care. J Midwifery Womens Health. 2019;64:98–103.

141. Posthumus AG, Borsboom GJ, Poeran J, Steegers EAP, Bonsel GJ. Geographical, ethnic and socio-economic differences in utilization of obstetric care in the Netherlands. PLoS One. 2016;11(6):e0156621.

142. Posthumus AG, Birnie E, van Veen MJ, Steegers EAP, Bonsel GJ. An antenatal prediction model for adverse birth outcomes in an urban population: the contribution of medical and non-medical risks. Midwifery. 2016;38:78–86.

143. Prentice A, Walton SM. Outcome of pregnancies referred to a general practitioner maternity unit in a district hospital. BMJ. 1989;299:1090–2.

144. Quinn EK, Noble J, Seale H, Ward JE. Provision of maternity care for women in remote Far West New South Wales: how far have we come? Aust J Rural Health. 2014;22:114–20.

145. Radomsky NA. Family practice obstetrics in a community hospital. Can Fam Physician. 1995;41:617–24.

146. Ravelli ACJ, Eskes M, Tromp M, Van Huis AM, Steegers EAP, Tamminga P, Bonsel GJ. Perinatale sterfte in Nederland gedurende 2000-2006; risicofactoren en risicoselectie [Perinatal mortality in The Netherlands 2000-2006; risk factors and risk selection]. Ned Tijdschr voor Geneeskd. 2008;152:2728–33.

147. Reddy K, Reginald P, Spring J, Nunn L, Mishra N. A free-standing low-risk maternity unit in the United Kingdom: does it have a role? J Obstet Gynaecol (Lahore). 2004;24(4):360–6.

148. Reilly SE, Brennecke SP, Smith J, Stewart MJ, Boland RA. Clinical features and outcomes of pregnancies complicated by pre-ecplampsia necessitating in-utero transfer. Pregnancy Hypertens. 2018;14:162–7.

149. Reither M, Germano E, DeGrazia M. Midwifery management of pregnant women who are obese. J Midwifery Womens Health. 2018;63:273–82.

150. Ressl B, O’Beirne M. Detecting breech presentation before labour: lessons from a low-risk maternity clinic. J Obstet Gynaecol Canada. 2015;37(8):702–6.

151. Reynolds J., Yudkin PL, Bull MJV. General practitioner obstetrics: does risk prediction work? J R Coll Gen Pract. 1988;38:307–10.

152. Richardson DK, Gabbe SG, Wind Y. Decision analysis of high-risk patient referral. Obstet Gynecol. 1984;63:496–501.

153. Richardson D, Rosoff A, Mcmenamin JP. Referral practices and health care costs: the dilemma of high risk obstetrics. J Leg Med. 1985;6(4):427–64.

154. Rijnders M, Baston H, Schönbeck Y, van der Pal K, Prins M, Green J, Buitendijk S. Perinatal factors related to negative or positive recall of birth experience in women 3 years postpartum in the Netherlands. Birth. 2008;35(2):107–16.

155. Roberts, Christine L, Henderson-Smart D, Ellwood DA, The High Risk Obstetric and Perinatal Advisory Working Group. Antenatal transfer of rural women to perinatal centres. Aust New Zeal J Obstet Gynaecol. 2000;40:377–84.

156. Rogers C, Pickersgill J, Palmer J, Broadbent M. Informing choices:outcomes for women at a stand-alone birth centre. Br J Midwifery. 2010;18(1):8–15.

157. Romijn A, Muijtjens AMM, de Bruijne MC, Donkers HHLM, Wagner C, de Groot CJM, Teunissen PW. What is normal progress in the first stage of labour? A vignette study of similarities and differences between midwives and obstetricians. Midwifery. 2016;41:104–9.

158. Rooks JP, Weatherby NL, Ernst EKM, Stapleton S, Rosen D, Rosenfield A. Outcomes of care in birth centers. The National Birth Center Study. N Engl J Med. 1989;321(26):1804–11.

159. Rooks JP, Weatherby NL, Ernst EKM. The National Birth Center Study part I — methodology and prenatal care and Rrferrals. J Nurse Midwifery. 1992;37(4):222–53.

160. Rooks JP, Weatherby NL, Ernst EKM. The National Birth Center Study part III — intrapartum and immediate postpartum and neonatal complications and transfers, postpartum and neonatal care, outcomes, and client satisfaction. J Nurse Midwifery. 1992;37(6):361–97.

161. Rosenblatt. Outcomes of regionalized perinatal care in Washington State. West J Med. 1988;149(1):98–102.

162. Rowe R, Fitzpatrick R, Hollowell J, Kurinczuk J. Transfers of women planning birth in midwifery units: data from the Birthplace prospective cohort study. BJOG. 2012;119:1081–90.

163. Rowe R, Li Y, Knight M, Brocklehurst P, Hollowell J. Maternal and perinatal outcomes in women planning vaginal birth after caesarean (VBAC) at home in England: secondary analysis of the Birthplace national prospective cohort study. BJOG. 2016;123:1123–32.

164. Ryan TD, Kidd GM. Maternal morbidity associated with in utero transfer. BMJ. 1989 Dec 2;299:1383–5.

165. Scherjon S. A comparison between the organization of obstetrics in Denmark and The Netherlands. Br J Obstet Gynaecol. 1986;93:684–9.

166. Scherman S, Smith J, Davidson M. The first year of a midwifery-led model of care in Far North Queensland. Med J Aust. 2008;188:85–8.

167. Schmidt N, Abelsen B, Oian P. Deliveries in maternity homes in Norway: results from a 2-year prospective study. Acta Obstet Gynecol Scand. 2002;81:731–7.

168. Schuit E, Hukkelhoven CWPM, van der Goes BY, Overbeeke I, Moons KGM, Mol BWJ, Groenwold RHH, Kwee A. Risk indicators for referral during labor from community midwife to gynecologist: a prospective cohort study. J Matern Neonatal Med. 2015;29(20):3304–11.

169. Schwartz RM, Muri JH, Overpeck MD, Pezzullo JC, Kogan MD. Use of high-technology care among women with high-risk pregnancies in the United States. Matern Child Health J. 2000;4(1):7–18.

170. Scupholme A, McLeod AGW, Robertson EG. A birth center affiliated with the tertiary care center: comparison of outcome. Obstet Gynecol. 1986;67(4):598–603.

171. Shaw R, Kitzinger C. Calls to a home birth helpline: empowerment in childbirth. Soc Sci Med. 2005;61:2374–83.

172. Shenai JP, Major CW, Gaylord MS, Blake WW, Simmons A, Oliver S, DeArmond D. A successful decade of regionalized perinatal care tennessee: the neonatal experience. J Perinatol. 1991;11(2):137–43.

173. Sidhu H, Heasley RN, Patterson CC, Halliday HL, Thompson W. Short term outcome in babies refused perinatal intensive care. BMJ. 1989 Sep 9;299:647–9.

174. Sloan EP, Kirsh S. Characteristics of obstetrical inpatients referred to a consultation-liaison psychiatry service in a tertiary-level university hospital. Arch Womens Ment Health. 2008;11:327–33.

175. Smit Y, Scherjon SA, Treffers PE. Elderly nulliparae in midwifery care in Amsterdam. Midwifery. 1997;13:73–7.

176. Smit Y, Scherjon S., Knuist M, Treffers P. Obstetric outcome of elderly low-risk nulliparae. Int J Gynecol Obstet. 1998;63:7–14.

177. Smit M, Ganzeboom A, Dawson JA, Walther FJ, Bustraan J, van Roosmalen JJM, te Pas AB. Feasibility of pulse oximetry for assessment of infants born in community based midwifery care. Midwifery. 2014;30:539–43.

178. Snowden JM, Tilden EL, Snyder J, Quigley B, Caughey AB, Cheng YW. Planned out-of-hospital birth and birth outcomes. N Engl J Med. 2015;373(27):2642–53.

179. Stern C, Permezel M, Petterson C, Lawson J, Eggers T, Kloss M. The Royal Women’s Hospital Family Birth Centre: the first 10 years reviewed. Aust New Zeal J Obstet Gynaecol. 1992;32(4):291–6.

180. Stewart MJ, Smith J, Boland RA. Optimizing outcomes in regionalized perinatal care: integrating maternal and neonatal emergency referral, triage, and transport. Curr Treat Options Pediatr. 2017;3:313–26.

181. Stolp I, Smit M, Luxemburg S, van den Akker T, de Waard J, van Roosmalen J, de Vos R. Ambulance transfer in case of postpartum hemorrhage after birth in primary midwifery care in the Netherlands: a prospective cohort study. Birth. 2015;42(3):227–34.

182. Street P, Gannon MJ, Holt EM. Community obstetric in West Berkshire. BMJ. 1991;302:698–700.

183. Strobino DM, Beth Silver G, Allston AA, Grason HA. Local health department perspectives on linkages among birthing hospitals. J Perinatol. 2003;23:610–9.

184. Styles M, Cheyne H, O’Carroll R, Greig F, Dagge-Bell F, Niven C. The Scottish Trial of Refer or Keep (the STORK study): midwives’intrapartum decision making. Midwifery. 2011;27:104–11.

185. Sullivan NH, Witte M. Care of the at-risk neonate born at home — a model for nurse-midwife/physician collaboration. J Nurse Midwifery. 1995;40(6):534–40.

186. Suzuki S, Satomi M, Miyake H. Referrals during labor in midwifery care. J Nippon Med Sch. 2009;74(4):226–8.

187. Suzuki S. Trend analysis of primary midwife-led delivery care at a Japanese perinatal center. Int J Med Sci. 2014;11:466–70.

188. Symon A, Winter C, Donnan PT, Kirkham M. Examining autonomy’s boundaries: a follow-up review of perinatal mortality cases in UK independent midwifery. Birth. 2010;37(4):280–7.

189. Tilyard MW, Seddon RJ, Oakley W, Murdoch CJ. Is outcome for general practitioner obstetricians influenced by workload and locality? N Z Med J. 1988 Apr 27;101:207–9.

190. Tromp M, Eskes M, Reitsma JB, Erwich JJHM, Brouwers HAA, Rijninks-van Driel GC, Bonsel GJ, Ravelli ACJ. Regional perinatal mortality differences in the Netherlands; care is the question. BMC Public Health. 2009 Dec 14;9:102.

191. Tucker J. Guidelines and management of mild hypertensive conditions in pregnancy in rural general practices in Scotland: issues of appropriateness and access. Qual Saf Heal Care. 2003;12:286–90.

192. Tucker J, McVicar A, Pitchforth E, Farmer J, Bryers H. Maternity care models in a remote and rural network: assessing clinical appropriateness and outcome indicators. Qual Saf Heal Care. 2010;19:83–9.

193. Van Alten D, Eskes E, Treffers PE. Midwifery in the Netherlands. The Wormerveer study; selection, mode of delivery, perinatal mortality and infant morbidity. BJOG. 1989;96:656–62.

194. van der Kooy J, de Graaf JP, Birnie DE, Denktas S, Steegers EAP, Bonsel GJ. Different settings of place of midwife-led birth: evaluation of a midwife-led birth centre. Springerplus. 2016;5:786.

195. van Haaren KMA, Springer MP. De kwaliteit van het verloskundig handelen van de huisarts [The quality of obstetric care given by the general practitioner]. Huisarts Wet. 2002;45(11):586–91.

196. Van Otterloo LR, Connelly CD. Risk-appropriate care to improve practice and birth outcomes. J Obstet Gynecol Neonatal Nurs. 2018;47:661–72.

197. van Stenus CMV, Gotink M, Boere-Boonekamp MM, Sools A, Need A. Through the client’s eyes: using narratives to explore experiences of care transfers during pregnancy, childbirth, and the neonatal period. BMC Pregnancy Childbirth. 2017;17:182.

198. van Stenus CMV, Boere-Boonekamp MM, Kerkhof EFGM, Need A. Client experiences with perinatal healthcare for high-risk and low-risk women. Women and Birth. 2018;31:e380–8.

199. van Wagner V, Osepchook C, Harney E, Crosbie C, Tulugak M. Remote midwifery in Nunavik, Québec, Canada: outcomes of perinatal care for the Inuulitsivik health centre, 2000-2007. Birth. 2012 Sep;39(3):230–7.

200. Vause S, Clarke B. Risk stratification and hierarchy of antenatal care. Best Pract Res Clin Obstet Gynaecol. 2014;28:483–94.

201. Vedam S, Goff M, Marnin VN. Closing the theory–practice gap: intrapartum midwifery management of planned homebirths. J Midwifery Womens Health. 2007;52(3):291–300.

202. Viisainen K, Gissler M, Hemminki E. Birth outcomes by level of obstetric care in Finland: a catchment area based analysis. J Epidemiol Community Heal. 1994;48:400–5.

203. Vos AA, van Voorst SF, Posthumus AG, Waelput AJM, Denktaş S, Steegers EAP. Process evaluation of the implementation of scorecard-based antenatal risk assessment, care pathways and interdisciplinary consultation: the Healthy Pregnancy 4 All study. Public Health. 2017;150:112–20.

204. Waldenström U, Nilsson C-A. A randomized controlled study of birth center care versus standard maternity care: effects on women’s health. Birth. 1997;24(1):17–26.

205. Waldenström U, Nilsson C-A, Winbladh B. The Stockholm Birth Centre Trial: maternal and infant outcome. Br J Obstet Gynaecol. 1997;104:410–8.

206. Wallace EM, Mackintosh CL, Brownlee M, Laidlaw L, Johnstone FD. A study of midwife-medical staff interaction in a labour ward environment. J Obstet Gynaecol (Lahore). 1995;15(3):165–70.

207. Wiegers TA, van der Zee J, Keirse MJNC. Transfer from home to hospital: what is its effect on the experience of childbirth? Birth. 1998;25(1):19–24.

208. Woodcock HC, Read AW, Moore DJ, Stanely FJ, Bower C. Planned homebirths in Western Australia 1981-1987: a descriptive study. Med J Aust. 1990 Dec;153:672–8.

209. Woodhart L, Goldstone J, Hartz D. The stories of women who are transferred due to threat of preterm birth. Women and Birth. 2018;31:307–12.

210. Wright JD, Silver RM, Bonanno C, Gaddipati S, Lu Y-S, Simpson LL, Herzog TJ, Schulkin J, D’Alton ME. Practice patterns and knowledge of obstetricians and gynecologists regarding placenta accreta. J Matern Neonatal Med. 2013;26(16):1602–9.
