# Supplementary material for: Towards a better understanding of risk selection in maternal and newborn care: A systematic scoping review
Source: PLoS One. 2020 Jun 8;15(6):e0234252. doi: 10.1371/journal.pone.0234252 (PMC7279596; doi:10.1371/journal.pone.0234252)
Supplement: S5 Table — (DOCX) [file pone.0234252.s005.docx]

**S3 List of included references**

| **Reference ID** | **First author** | **Year of publication** | **Country**  Country studied. If not applicable, country first author |
| --- | --- | --- | --- |
| [1] | Aalfs | 2003 | Netherlands |
| [2] | Accortt | 2017 | USA |
| [3] | Allen | 2007 | Ireland |
| [4] | Amelink-Verburg | 2008 | Netherlands |
| [5] | Amelink-Verburg | 2009 | Netherlands |
| [6] | Amelink-Verburg | 2010 | Netherlands |
| [7] | Ammari | 1995 | England |
| [8] | Anderson | 1995 | USA |
| [9] | Badgery-Parker | 2012 | Australia |
| [10] | Bahry | 1989 | USA |
| [11] | Baird | 1996 | Scotland |
| [12] | Bais | 2004 | Netherlands |
| [13] | Baker | 1992 | USA |
| [14] | Baldwin | 1995 | USA |
| [15] | Baldwin | 2019 | Australia |
| [16] | Bernitz | 2011 | Norway |
| [17] | Bernstein | 2016 | USA |
| [18] | Binders | 2011 | USA |
| [19] | Biro | 1991 | Australia |
| [20] | Blix | 2016 | Norway, Sweden, Denmark and Iceland |
| [21] | Blondel | 2009 | 9 European countries  Belgium, Denmark, France, Germany, Italy, Netherlands, Poland, Portugal and UK (England, Ireland, Scotland, Wales) |
| [22] | Bode | 2001 | USA |
| [23] | Bodner | 2017 | Austria |
| [24] | Bolbocean | 2016 | Canada |
| [25] | Bovbjerg | 2017 | USA |
| [26] | Britt | 2006 | USA |
| [27] | Bronstein | 2011 | USA |
| [28] | Butler | 2015 | Ireland |
| [29] | Carolan | 2009 | Canada |
| [30] | Chambliss | 1992 | USA |
| [31] | Cohen | 2015 | France |
| [32] | Cooke | 2004 | Australia |
| [33] | Cordero | 1989 | USA |
| [34] | Craig | 1985 | USA |
| [35] | Crotty | 1990 | Australia |
| [36] | Daemers | 2014 | Netherlands |
| [37] | David | 2006 | Germany |
| [38] | De Galan-Roosen | 1999 | Netherlands |
| [39] | De Jonge | 2009 | Netherlands |
| [40] | De Jonge | 2011 | Netherlands |
| [41] | De Jonge | 2013 | Netherlands |
| [42] | De Jonge | 2015 | Netherlands |
| [43] | De Reu | 2010 | Netherlands |
| [44] | De Weerd | 2001 | Netherlands |
| [45] | DeJong | 1981 | USA |
| [46] | Delaney-Black | 1989 | USA |
| [47] | Dencker | 2017 | Ireland |
| [48] | Dijkstra | 2003 | Netherlands |
| [49] | Donohue | 2009 | USA |
| [50] | Eden | 2005 | USA |
| [51] | Eide | 2009 | Norway |
| [52] | Engjom | 2018 | Norway |
| [53] | Eskes | 2017 | Netherlands |
| [54] | Evers | 2010 | Netherlands |
| [55] | Evers | 2013 | Netherlands |
| [56] | Ferndale | 2017 | Australia |
| [57] | Ferrazzi | 2015 | Italy |
| [58] | Finnström | 2006 | Sweden |
| [59] | Fleissig | 1996 | England |
| [60] | Ford | 1991 | England |
| [61] | Fullerton | 1997 | USA |
| [62] | Garite | 1995 | USA |
| [63] | Gaudineau | 2013 | France |
| [64] | Geerts | 2014 | Netherlands |
| [65] | George | 2018 | Australia |
| [66] | Giles | 2000 | Australia |
| [67] | Gillespie | 2018 | Ireland |
| [68] | Godbole | 2013 | England |
| [69] | Goh | 2015 | Australia |
| [70] | Govaerts | 2017 | Netherlands |
| [71] | Grigg | 2015 | New Zealand |
| [72] | Gyte | 2009 | England and Wales |
| [73] | Hein | 1986 | USA |
| [74] | Hemmeniki | 1990 | Finland |
| [75] | Hemminiki | 2011 | Finland |
| [76] | Hollingworth | 2018 | Australia |
| [77] | Holt | 2001 | Norway |
| [78] | Hopkins | 2018 | USA |
| [79] | Hueston | 1994 | USA |
| [80] | Humphrey | 2017 | Australia |
| [81] | Hundley | 1994 | Scotland |
| [82] | Hutchinson | 2014 | Australia |
| [83] | Hutton | 2009 | Canada |
| [84] | Jackson | 2006 | England |
| [85] | James | 2017 | UK (England, Ireland, Scotland, Wales) |
| [86] | Janssen | 2003 | Canada |
| [87] | Jeffery | 2017 | England |
| [88] | Jordan | 1995 | Canada |
| [89] | Kim | 2010 | USA |
| [90] | Kirke | 2010 | Australia |
| [91] | Knight | 2018 | UK (England, Ireland, Scotland, Wales) |
| [92] | Knox | 1984 | USA |
| [93] | Kollée | 1998 | Netherlands |
| [94] | Koshida | 2015 | Japan |
| [95] | Kruske | 2015 | Australia |
| [96] | Kruske | 2016 | Australia |
| [97] | Kuliukas | 2015 | Australia |
| [98] | Kwong | 2018 | USA |
| [99] | Lagendijk | 2018 | Netherlands |
| [100] | Lalor | 2007 | Ireland |
| [101] | Laube | 1983 | USA |
| [102] | Lavender | 2006 | England |
| [103] | Law | 1999 | China |
| [104] | Leddy | 2011 | USA |
| [105] | Lennox | 1992 | Scotland |
| [106] | Lessaris | 2002 | USA |
| [107] | Lim | 2017 | England |
| [108] | Lubchenco | 1989 | USA |
| [109] | Lundeen | 2016 | USA |
| [110] | Maassen | 2008 | Netherlands |
| [111] | MacVicar | 1993 | England |
| [112] | Magann | 2012 | USA |
| [113] | Mansbridge | 2014 | England |
| [114] | Marlow | 2014 | England |
| [115] | Martijn | 2013 | Netherlands |
| [116] | Matthey | 2016 | Scotland |
| [117] | Mayer | 2018 | England |
| [118] | McCool | 2015 | USA |
| [119] | McIntyre | 2012 | Australia |
| [120] | McMurtrie | 2009 | Australia |
| [121] | Mengel | 1987 | USA |
| [122] | Meuli | 1984 | USA |
| [123] | Mito | 2015 | Japan |
| [124] | Monk | 2014 | Australia |
| [125] | Montgomery-Andersen | 2010 | Greenland |
| [126] | Morano | 2007 | Italy |
| [127] | Morley | 2018 | England |
| [128] | Morriss | 2013 | USA |
| [129] | Morriss | 2018 | USA |
| [130] | Nuovo | 1985 | USA |
| [131] a | Offerhaus | 2015 | Netherlands |
| [132] b | Offerhaus | 2015 | Netherlands |
| [133] c | Offerhaus | 2015 | Netherlands |
| [134] | Papiernik | 1995 | France |
| [135] | Patterson | 2017 | New Zealand |
| [136] | Peddle | 1983 | Canada |
| [137] | Perdok | 2015 | Netherlands |
| [138] | Perdok | 2016 | Netherlands |
| [139] | Persson | 2004 | Greenland |
| [140] | Phillippi | 2019 | USA |
| [141] a | Posthumus | 2016 | Netherlands |
| [142] b | Posthumus | 2016 | Netherlands |
| [143] | Prentice | 1989 | England |
| [144] | Quinn | 2014 | Australia |
| [145] | Radomsky | 1995 | Canada |
| [146] | Ravelli | 2008 | Netherlands |
| [147] | Reddy | 2004 | England |
| [148] | Reilly | 2018 | Australia |
| [149] | Reither | 2018 | USA |
| [150] | Ressl | 2015 | Canada |
| [151] | Reynolds | 1988 | England |
| [152] | Richardson | 1984 | USA |
| [153] | Richardson | 1985 | USA |
| [154] | Rijnders | 2008 | Netherlands |
| [155] | Roberts | 2000 | Australia |
| [156] | Rogers | 2010 | England |
| [157] | Romijn | 2016 | Netherlands |
| [158] | Rooks | 1989 | USA |
| [159] | Rooks | 1992 | USA |
| [160] | Rooks | 1992 | USA |
| [161] | Rosenblatt | 1988 | USA |
| [162] | Rowe | 2012 | England |
| [163] | Rowe | 2016 | England |
| [164] | Ryan | 1989 | England |
| [165] | Scherjon | 1986 | Netherlands and Denmark |
| [166] | Scherman | 2008 | Australia |
| [167] | Schmidt | 2002 | Norway |
| [168] | Schuit | 2015 | Netherlands |
| [169] | Schwartz | 2000 | USA |
| [170] | Scupholme | 1986 | USA |
| [171] | Shaw | 2005 | UK (England, Ireland, Scotland, Wales) |
| [172] | Shenai | 1991 | USA |
| [173] | Sidhu | 1989 | Ireland |
| [174] | Sloan | 2008 | Canada |
| [175] | Smit | 1997 | Netherlands |
| [176] | Smit | 1998 | Netherlands |
| [177] | Smit | 2014 | Netherlands |
| [178] | Snowden | 2016 | USA |
| [179] | Stern | 1992 | Australia |
| [180] | Stewart | 2017 | Australia |
| [181] | Stolp | 2015 | Netherlands |
| [182] | Street | 1991 | England |
| [183] | Strobino | 2003 | USA |
| [184] | Styles | 2011 | Scotland |
| [185] | Sullivan | 1995 | USA |
| [186] | Suzuki | 2009 | Japan |
| [187] | Suzuki | 2014 | Japan |
| [188] | Symon | 2010 | UK (England, Ireland, Scotland, Wales) |
| [189] | Tilyard | 1988 | New Zealand |
| [190] | Tromp | 2009 | Netherlands |
| [191] | Tucker | 2003 | Scotland |
| [192] | Tucker | 2010 | Scotland |
| [193] | Van Alten | 1989 | Netherlands |
| [194] | Van der Kooy | 2016 | Netherlands |
| [195] | Van Haaren | 2002 | Netherlands |
| [196] | Van Otterloo | 2018 | USA |
| [197] | Van Stenus | 2017 | Netherlands |
| [198] | Van Stenus | 2018 | Netherlands |
| [199] | Van Wagner | 2012 | Canada |
| [200] | Vause | 2014 | USA |
| [201] | Vedam | 2007 | Canada |
| [202] | Viisainen | 1994 | Finland |
| [203] | Vos | 2017 | Netherlands |
| [204] | Waldenström | 1997 | Sweden |
| [205] | Waldenström | 1997 | Sweden |
| [206] | Wallace | 1995 | Scotland |
| [207] | Wiegers | 1998 | Netherlands |
| [208] | Woodcock | 1990 | Australia |
| [209] | Woodhart | 2018 | Australia |
| [210] | Wright | 2013 | USA |

1. Aalfs CM, Smets EMA, de Haes HCJM, Leschot NJ. Referral for genetic counselling during pregnancy: limited alertness and awareness about genetic risk factors among GPs. Fam Pract. 2003;20(2):135–41.

2. Accortt EE, Wong MS. It Is Time for Routine Screening for Perinatal Mood and Anxiety Disorders in Obstetrics and Gynecology Settings. Obstet Gynecol Surv. 2017;72(9):553–68.

3. Allen C, Greene R, Higgins J. Audit of antenatal clinic for high-risk obstetric patients; activity and outcomes. Ir Med J. 2007;100(9):591–3.

4. Amelink-Verburg MP, Verloove-Vanhorick SP, Hakkenberg RMA, Veldhuijzen IME, Bennebroek Gravenhorst J, Buitendijk SE. Evaluation of 280 000 cases in Dutch midwifery practices: a descriptive study. BJOG. 2008;115:570–8.

5. Amelink-Verburg MP, Rijnders MEB, Buitendijk SE. A trend analysis in referrals during pregnancy and labour in Dutch midwifery care 1988-2004. BJOG. 2009;116:923–32.

6. Amelink-Verburg MP, Buitendijk SE. Pregnancy and labour in the Dutch maternity care system: what is normal? The role division between midwives and obstetricians. J Midwifery Womens Health. 2010;55(3):216–25.

7. Ammari F, Gregory R. Screening for gestational diabetes in a population at high risk. Pract Diabetes Int. 1995;13(5):150–2.

8. Anderson RE, Murphy PA. Outcomes of 11,788 planned home births attended by certified nurse-midwives. A retrospective descriptive study. J Nurse Midwifery. 1995;40(6):483–92.

9. Badgery-Parker T, Ford JB, Jenkins MG, Morris JM, Roberts CL. Patterns and outcomes of preterm hospital admissions during pregnancy in NSW, 2001–2008. Med J Aust. 2012;196(4):261–5.

10. Bahry VJ, Fullerton JT, Lops VR. Provision of comprehensive perinatal services through rural outreach: a model program. J Rural Heal. 1989;5(4):387–96.

11. Baird AG, Jewell D, Walker J. Management of labour in an isolated rural maternity hospital. BMJ. 1996;312:223–6.

12. Bais JMJ, Eskes M, Pel M, Bonsel GJ, Bleker OP. Effectiveness of detection of intrauterine growth retardation by abdominal palpation as screening test in a low risk population: an observational study. Eur J Obstet Gynecol Reprod Biol. 2004;116:164–9.

13. Baker SL, Kronenfeld JJ. High risk channeling to improve medicaid maternal and infant care. J Health Soc Policy. 1992;3(4):29–49.

14. Baldwin L-M, Hart G, Lloyd M, Fordyce M, Rosenblatt RA. Defensive medicine and obstetrics. JAMA J Am Med Assoc. 1995 Nov;274(20):1606–10.

15. Baldwin A, Harvey C, Willis E, Ferguson B, Capper T. Transitioning across professional boundaries in midwifery models of care: a literature review. Women and Birth. 2019;32:195–203.

16. Bernitz S, Rolland R, Blix E, Jacobsen M, Sjøborg K, Øian P. Is the operative delivery rate in low-risk women dependent on the level of birth care? A randomised controlled trial. BJOG. 2011;118:1357–64.

17. Bernstein JA, McCloskey L, Gebel CM, Iverson RE, Lee-Parritz A. Lost opportunities to prevent early onset type 2 diabetes mellitus after a pregnancy complicated by gestational diabetes. BMJ Open Diabetes Res Care. 2016 Jun 17;4:e000250.

18. Binder S, Hill K, Meinzen-Derr J, Greenberg JM, Narendran V. Increasing VLBW deliveries at subspecialty perinatal centers via perinatal putreach. Pediatrics. 2011;127(3):487–93.

19. Biro M, Lumley J. The safety of team midwifery: the first decade of the Monash Birth Centre. Med J Aust. 1991;155:478–80.

20. Blix E, Kumle MH, Ingversen K, Huitfeldt AS, Hegaard HK, Ólafsdóttir ÓÁ, Øian P, Lindgren H. Transfers to hospital in planned home birth in four Nordic countries - a prospective cohort study. Acta Obstet Gynecol Scand. 2016;95:420–8.

21. Blondel B, Papiernik E, Delmas D, Künzel W, Weber T, Maier R, Kollée L, Zeitlin J. Organisation of obstetric services for very preterm births in Europe: results from the MOSAIC project. BJOG. 2009 Sep;116(10):1364–72.

22. Bode MM, O’Shea MT, Metzguer KR, Stiles AD. Perinatal regionalization and neonatal mortality in North Carolina, 1968-1994. Am J Obstet Gynecol. 2001;184(6):1302–7.

23. Bodner-Adler B, Kimberger O, Griebaum J, Husslein P, Bodner K. A ten-year study of midwife-led care at an Austrian tertiary care center: a retrospective analysis with special consideration of perineal trauma. BMC Pregnancy Childbirth. 2017;17:357.

24. Bolbocean C, Wintermark P, Shevell MI, Oskoui M. Perinatal regionalization and implications for long-term health outcomes in cerebral palsy. Can J Neurol Sci / J Can des Sci Neurol. 2016;1–6.

25. Bovbjerg ML, Cheyney M, Brown J, Cox KJ, Leeman L. Perspectives on risk: Assessment of risk profiles and outcomes among women planning community birth in the United States. Birth. 2017;44:209–21.

26. Britt DW, Eden RD, Evans MI. Matching risk and resources in high-risk pregnancies. J Matern Neonatal Med. 2006;19(10):645–50.

27. Bronstein JM, Ounpraseuth S, Jonkman J, Lowery CL, Fletcher D, Nugent RR, Hall RW. Improving Perinatal Regionalization for Preterm Deliveries in a Medicaid Covered Population: Initial Impact of the Arkansas ANGELS Intervention. Health Serv Res. 2011;46(4):1082–103.

28. Butler MM, Sheehy L, Kington MM, Walsh MC, Brosnan MC, Murphy M, Naughton C, Drennan J, Barry T. Evaluating midwife-led antenatal care: Choice, experience, effectiveness, and preparation for pregnancy. Midwifery. 2015;31:418–25.

29. Carolan M, Hodnett E. Discovery of soft markers on fetal ultrasound: maternal implications. Midwifery. 2009;25:654–64.

30. Chambliss LR, Daly C, Medearis AL, Ames M, Kayne M, Paul R. The role of selection bias in comparing cesarean birth rates between physician and midwifery management. Obstet Gynecol. 1992;80(2):161–5.

31. Cohen L, Schaeffer M, Davideau J-L, Tenenbaum H, Huck O. Obstetric knowledge, attitude, and behavior concerning periodontal diseases and treatment needs in pregnancy: influencing factors in France. J Periodontol. 2015;86(3):398–405.

32. Cooke HM, Waters DL, Dyer K, Lawler J, Picone D. Development of a best practice model of midwifery-led antenatal care. Aust Midwifery. 2004;17(2):21–5.

33. Cordero L, Schurman S, Zuspan FP. Appropriateness of antenatal referrals to a regional perinatal center. J Perinatol. 1989;9(1):38–42.

34. Craig AS, Berg AO, Kirkwood CR. Obstetrie consultations during labor and delivery in a university based family practice. J Fam Pract. 1985;20(5):481–5.

35. Crotty M, Ramsay AT, Smart R, Chan A. Planned homebirths in South Australia 1976-1987. Med J Aust. 1990;153:664–71.

36. Daemers DOA, Wijnen HAA, van Limbeek EBM, Budé LM, Nieuwenhuijze MJ, Spaanderman MEA, De Vries RG. The impact of obesity on outcomes of midwife-led pregnancy and childbirth in a primary care population: a prospective cohort study. BJOG. 2014;121:1403–14.

37. David M, Berg G, Werth I, Pachaly J, Mansfeld A, Kentenich H. Intrapartum transfer from a birth centre to a hospital – reasons, procedures, and consequences. Acta Obstet Gynecol Scand. 2006;85:422–8.

38. de Galan-Roosen AEM, Kuijpers JC, Mackenbach JP. Perinatal mortality in Delft and environs, 1983 - 1992: further decrease possible by specific attention to lethal congenital anomalies and placental insufficiency. Ned Tijdschr voor Geneeskd. 1999;143:152–7.

39. de Jonge A, van der Goes BY, Ravelli ACJ, Amelink-Verburg MP, Mol BW, Nijhuis JG, Gravenhorst JB, Buitendijk SE. Perinatal mortality and morbidity in a nationwide cohort of 529 688 low-risk planned home and hospital births. BJOG. 2009;116:1177–84.

40. de Jonge A, Rijnders M, Agyemang C, van der Stouwe R, den Otter J, Van den Muijsenbergh METC, Buitendijk S. Limited midwifery care for undocumented women in the Netherlands. J Psychosom Obstet Gynecol. 2011;32(4):182–8.

41. de Jonge A, Mesman JAJM, Mannien J, Zwart JJ, van Dillen J, van Roosmalen J. Severe adverse maternal outcomes among low risk women with planned home versus hospital births in the Netherlands: nationwide cohort study. BMJ. 2013;346:f3263.

42. de Jonge A, Mesman JAJM, Manniën J, Zwart JJ, Buitendijk SE, van Roosmalen J, van Dillen J. Severe adverse maternal outcomes among women in midwife-led versus obstetrician-led care at the onset of labour in the Netherlands: a nationwide cohort study. PLoS One. 2015;10(5):e0126266.

43. De Reu PAOM, Oosterbaan HP, Smits LJM, Nijhuis JG. Avoidable mortality in small-for-gestational-age children in the Netherlands. J Perinat Med. 2010;38(3):311–8.

44. de Weerd S, Wouters MGAJ, Mom-Boertjens J, Bos KL. Preconception advice: evaluation of an outpatients’ clinic at a university hospital. Nederlands Tijdschrift voor de Geneeskunde. 2001.

45. DeJong RN, Carr KC. An out-of-hospital birth center using university referral. Obstet Gynecol. 1981 Dec;58(6):703–7.

46. Delaney-Black V, Lubchenco LO, Joseph Butterfield L, Goldson E, Koops BL, Lazotte DC. Outcome of very-low-birth-weight infants: are populations of neonates inherently different after antenatal versus neonatal referral? Am J Obstet Gynecol. 1989;160(3):545–52.

47. Dencker A, Smith V, McCann C, Begley C. Midwife-led maternity care in Ireland – a retrospective cohort study. BMC Pregnancy Childbirth. 2017;17:101.

48. Dijkstra K, Kuyvenhoven M, Verheij T, Iedema H, Springer M, Visser G. Dreigende vroeggeboorte; opvattingen en werkwijze van verloskundigen, huisartsen en gynaecologen [Threatened pre-term delivery: opinions and working methods of midwives, GPs and gynaecologists]. Huisarts Wet. 2003;46(3):129–33.

49. Donohue PK, Boss RD, Shepard J, Graham E, Allen MC. Intervention at the border of viability – perspective over a decade. Arch Pediatr Adolesc Med. 2009 Oct;163(10):902–6.

50. Eden RD, Eden RD, Penka A, Britt DW, Landsberger EJ, Evans MI. Re-evaluating the role of the MFM specialist: lead, follow, or get out of the way. J Matern Neonatal Med. 2005;18(4):253–8.

51. Eide BI, Nilsen ABV, Rasmussen S. Births in two different delivery units in the same clinic – a prospective study of healthy primiparous women. BMC Pregnancy Childbirth. 2009;9:25.

52. Engjom HM, Morken N-H, Høydahl E, Norheim OF, Klungsøyr K. Risk of eclampsia or HELLP-syndrome by institution availability and place of delivery – a population-based cohort study. Pregnancy Hypertens. 2018;14:1–8.

53. Eskes M, Waelput AJM, Scherjon SA, Bergman KA, Abu-Hanna A, Ravelli ACJ. Small for gestational age and perinatal mortality at term: An audit in a Dutch national cohort study. Eur J Obstet Gynecol Reprod Biol. 2017;215:62–7.

54. Evers ACC, Brouwers HAA, Hukkelhoven CWPM, Nikkels PGJ, Boon J, van Egmond-Linden A, Hillegersberg J, Snuif YS, Sterken-Hooisma S, Bruinse HW, Kwee A. Perinatal mortality and severe morbidity in low and high risk term pregnancies in the Netherlands: prospective cohort study. BMJ. 2010;341:c5639.

55. Evers ACC, Brouwers HAA, Nikkels PGJ, Boon J, van Egmond-Linden A, Groenendaal F, Hart C, Hillegersberg J, Snuif YS, Sterken-Hooisma S, Steins Bisschop CN, Westerhuis MEMH, Bruinse HW, Kwee A. Substandard care in delivery-related asphyxia among term infants: prospective cohort study. Acta Obstet Gynecol Scand. 2013;92:85–93.

56. Ferndale D, Meuter RFI, Watson B, Gallois C. ‘You don’t know what’s going on in there’: a discursive analysis of midwifery hospital consultations. Health Risk Soc. 2017;19(7–8):411–31.

57. Ferrazzi E, Visconti E, Paganelli AM, Campi CM, Lazzeri C, Cirillo F, Livio S, Piola C. The outcome of midwife-led labor in low-risk women within an obstetric referral unit. J Matern Neonatal Med. 2015;28(13):1530–6.

58. Finnström O, Berg G, Norman A, Olausson PO. Size of delivery unit and neonatal outcome in Sweden. A catchment area analysis. Acta Obstet Gynecol Scand. 2006;85:63–7.

59. Fleissig A, Kroll D, McCarthy M. Is community-led maternity care a feasible option for women assessed at low risk and those with complicated pregnancies? Results of a population based study in South Camden, London. Midwifery. 1996;12:191–7.

60. Ford C, Iliffe S, Franklin O. Outcome of planned home births in an inner city practice. BMJ. 1991 Dec 14;303:1517–9.

61. Fullerton JT, Jackson D, Snell BJ, Besser M, Dickinson C, Garite T. Transfer rates from freestanding birth centers – a comparison with the National Birth Center Study. J Nurse Midwifery. 1997;42(1):9–16.

62. Garite TJ, Snell BJ, Walker DL, Darrow VC. Development and experience of a university based, freestanding birthing center. Obstet Gynecol. 1995 Sep;86(3):411–6.

63. Gaudineau A, Sauleau E-A, Nisand I, Langer B. Obstetric and neonatal outcomes in a home-like birth centre: a case – control study. Arch Gynecol Obstet. 2013;287:211–6.

64. Geerts Caroline C, Trudy K, Lagro-Janssen Antoine LM, Twisk Jos WR, Dillen V, Jeroen, Jonge D, Ank. Birth setting, transfer and maternal sense of control: results from the DELIVER study. BMC Pregnancy Childbirth. 2014;14:27.

65. George A, Dahlen HG, Blinkhorn A, Ajwani S, Bhole S, Ellis S, Yeo A, Elcombe E, Johnson M. Evaluation of a midwifery initiated oral health-dental service program to improve oral health and birth outcomes for pregnant women: a multi-centre randomised controlled trial. Int J Nurs Stud. 2018;82:49–57.

66. Giles W, Bisits A, Knox M, Madsen G, Smith R. The effect of fetal fibronectin testing on admissions to a tertiary maternal-fetal medicine unit and cost savings. Am J Obstet Gynecol. 2000;182:439–42.

67. Gillespie M, Sinclair M, Stockdale J, Bunting B, Condell J. Online educational resources for health professionals caring for pregnant women with heart disease: a scoping literature review using Arksey and O’Malley’s methodological framework. Evid Based Midwifery. 2018;16(2):55–61.

68. Godbole G, Irish D, Basarab M, Mahungu T, Fox-Lewis A, Thorne C, Jacobs M, Dusheiko G, Rosenberg WMC, Suri D, Millar AD, Nastouli E. Management of hepatitis B in pregnant women and infants: a multicentre audit from four London hospitals. BMC Pregnancy Childbirth. 2013;13:222.

69. Goh A, Browning Carmo K, Morris J, Berry A, Wall M, Abdel-Latif M. Outcomes of high-risk obstetric transfers in New South Wales and the Australian capital territory: the high-risk obstetric transfer study. Aust New Zeal J Obstet Gynaecol. 2015;1–6.

70. Govaerts L, Srebniak M, Diderich K, Joosten M, Riedijk S, Knapen M, Go A, Papatsonis D, de Graaf K, Toolenaar T, van der Steen S, Huijbregts G, Knijnenburg J, de Vries F, Van Opstal D, Galjaard R-J. Prenatal diagnosis of susceptibility loci for neurodevelopmental disorders - genetic counseling and pregnancy outcome in 57 cases. Prenat Diagn. 2017;37:73–80.

71. Grigg CP, Tracy SK, Tracy M, Schmied V, Monk A. Transfer from primary maternity unit to tertiary hospital in New Zealand – timing, frequency, reasons, urgency and outcomes: part of the Evaluating Maternity Units study. Midwifery. 2015;31:879–87.

72. Gyte G, Dodwell M, Newburn M, Sandall J, Macfarlane A, Bewley S. Estimating intrapartum-related perinatal mortality rates for booked home births: when the ‘best’ available data are not good enough. BJOG. 2009;116:933–42.

73. Hein HA, Burmeister LF. The effect of ten years of regionalized perinatal health care in Iowa, U.S.A. Eur J Obstet Gynecol Reprod Biol. 1986;21:33–48.

74. Hemminki E, Malin M, Kojo-Austin H. Prenatal care in Finland: from primary to tertiary health care? Int J Heal Serv. 1990;20(2):221–32.

75. Hemminki E, Heino A, Gissler M. Should births be centralised in higher level hospitals? Experiences from regionalised health care in Finland. BJOG. 2011;118:1186–95.

76. Hollingworth J, Pietsch R, Epee-Bekima M, Nathan E. Time to delivery: transfers for threatened preterm labour and prelabour rupture of membranes in Western Australia. Aust J Rural Health. 2018;26:42–7.

77. Holt J, Vold IN, Backe B, Johansen MV, Øian P. Child births in a modified midwife managed unit: Selection and transfer according to intended place of delivery. Acta Obstet Gynecol Scand. 2001;80:206–12.

78. Hopkins MK, Goldstein SA, Ward CC, Kuller JA. Evaluation and management of aternal congenital ceart disease: a review. Obstet Gynecol Surv. 2018;73(2):116–24.

79. Hueston WJ, The Factors Meeting Cesarean Section (FACS) Study Group. Obstetric referral in family practice. J Fam Pract. 1994 Apr;38(4):368–72.

80. Humphrey MD, Foxcroft KF, Callaway LK. Obstetric risk score – revalidated for triaging high-risk pregnancies in rural areas. Aust New Zeal J Obstet Gynaecol. 2017;57:63–7.

81. Hundley VA, Cruickshank FM, Lang GD, Glazener CMA, Milne JM, Turner M, Blyth D, Mollison J, Donaldson C. Midwife managed delivery unit: a randomised controlled comparison with consultant led care. BMJ. 1994 Nov 26;309(6966):1400–4.

82. Hutchinson FH, Davies MW. Time-to-delivery after maternal transfer to a tertiary perinatal centre. Biomed Res Int. 2014;325919.

83. Hutton EK, Reitsma AH, Kaufman K. Outcomes associated with planned home and planned hospital births in low-risk women attended by midwives in ontario, Canada, 2003-2006: a retrospective cohort study. Birth. 2009;36(3):180–9.

84. Jackson CJ, Bosio P, Habiba M, Waugh J, Kamal P, Dixon-Woods M. Referral and attendance at a specialist antenatal clinic: qualitative study of women’s views. BJOG. 2006;113:909–13.

85. James A, Endacott R, Stenhouse E. Maternity High Dependency Care (MHDC) in Obstetric Units remote from tertiary referral centres; findings of a modified Delphi study. Evid Based Midwifery. 2017;15(4):120–7.

86. Janssen PA, Lee SK, Ryan ER, Saxell L. An evaluation of process and protocols for planned home birth attended by regulated midwives in British Columbia. J Midwifery Womens Health. 2003;48(2):138–45.

87. Jeffery J, Hewison A, Goodwin L, Kenyon S. Midwives’ experiences of performing maternal observations and escalating concerns: a focus group study. BMC Pregnancy Childbirth. 2017;17:282.

88. Jordan JM, Gaspar D. Family practice obstetrics in a teaching hospital – Does a tertiary care environment make a dfference? Can Fam Physician. 1995;41(April):610–5.

89. Kim JJ, La Porte LM, Corcoran M, Magasi S, Batza J, Silver RK. Barriers to mental health treatment among obstetric patients at risk for depression. Am J Obstet Gynecol. 2010;202:312.e1-312.e5.

90. Kirke AB. How safe is GP obstetrics? An assessment of antenatal risk factors and perinatal outcomes in one rural practice. Rural Remote Health. 2010;10:1545.

91. Knight M. The findings of the MBRRACE-UK confidential enquiry into maternal deaths and morbidity. Obstet Gynaecol Reprod Med. 2018;29(1):21–3.

92. Knox GE, Schnitker KA. In-utero transport. Clin Obstet Gynecol. 1984;27(1):11–6.

93. Kollée LAA, Den Ouden AL, Drewes JG, Brouwers HAA, Verwey RA, Verloove-Vanhorick SP. Toename van perinatale verwijzing naar regionale centra bij vroeggeboorte in Nederland: vergelijking van 1983 en 1993 [Increased perinatal referral to regional centres of premature infants in the Netherlands: comparison of 1983 and 1993]. Ned Tijdschr voor Geneeskd. 1998;142(3):131–4.

94. Koshida S, Ono T, Tsuji S, Murakami T, Takahashi K. Recommendations for preventing stillbirth: a regional population-based study in Japan during 2007-2011. Tohoku J Exp Med. 2015;235:145–9.

95. Kruske S, Schultz T, Eales S, Kildea S. A retrospective, descriptive study of maternal and neonatal transfers, and clinical outcomes of a primary maternity unit in rural Queensland, 2009–2011. Women and Birth. 2015;28:30–9.

96. Kruske S, Kildea S, Jenkinson B, Pilcher J, Robin S, Rolfe M, Kornelsen J, Barclay L. Primary maternity units in rural and remote Australia: results of a national survey. Midwifery. 2016;40:1–9.

97. Kuliukas L, Hauck Y, Duggan R, Lewis L. The phenomenon of intrapartum transfer from a western Australian birth centre to a tertiary maternity hospital: the overall experiences of partners. Midwifery. 2015;31:e87–93.

98. Kwong AJ, Chang MS, Tuomala RE, Riley LE, Robinson JN, Mutinga ML, Andersson KL, Brown Jr. RS, Oken E, Ukomadu C, Rutherford AE. Peripartum care for mothers diagnosed with hepatitis B during pregnancy: a survey of provider practices. Matern Child Health J. 2018;22:1345–51.

99. Lagendijk J, Vos AA, Bertens LCM, Denktas S, Bonsel GJ, Steyerberg EW, Been J V., Steegers EAP. Antenatal non-medical risk assessment and care pathways to improve pregnancy outcomes: a cluster randomised controlled trial. Eur J Epidemiol. 2018;33:579–89.

100. Lalor JG, Devane D, Begley CM. Unexpected diagnosis of fetal abnormality: women’s encounters with caregivers. Birth. 2007;34(1):80–8.

101. Laube DW. Experience with an alternative birth center in a university hospital. J Reprod Med. 1983;391–6.

102. Lavender T, Alfirevic Z, Walkinshaw S. Effect of different partogram action lines on birth outcomes – a randomized controlled trial. Obstet Gynecol. 2006;108(2):295–302.

103. Law YYH, Lam K-Y. A randomized controlled Ttial comparing midwife-managed care and obstetrician-managed care for women assessed to be at low risk in the initial intrapartum period. J Obstet Gynaecol Res. 1999;25(2):107–12.

104. Leddy MA, Lawrence H, Schulkin J. Obstetrician-gynecologists and womens mental health: Findings of the collaborative ambulatory research network 2005-2009. Obstet Gynecol Surv. 2011;66(5):316–23.

105. Lennox CE. Transferring at-risk babies in-utero or neonatally: a decade’s experience from a peripheral consultant maternity unit. Health Bull (Raleigh). 1992;50(5):362–7.

106. Lessaris KJ, Annibale DJ, Southgate MW, Hulsey TC, Ohning BL. Effects of changing health care financial policy on very low birthweight neonatal outcomes. South Med J. 2002;95(4):426–30.

107. Lim JCES, Cauldwell M, Patel RR, Uebing A, Curry RA, Johnson MR, Gatzoulis MA, Swan L. Management of marfan syndrome during pregnancy: a real world experience from a joint cardiac obstetric service. Int J Cardiol. 2017;243:180–4.

108. Lubchenco LO, Joseph Butterfield L, Delaney-Black V, Goldson E, Koops BL, Lazotte DC. Outcome of very-low-birth-weight infants: does antepartum versus neonatal referral have a better impact on mortality, morbidity, or long-term outcome? Am J Obstet Gynecol. 1989;160(3):539–45.

109. Lundeen T. Intrapartum and Postpartum Transfers to a Tertiary Care Hospital from Out-of-Hospital Birth Settings: A Retrospective Case Series. J Midwifery Womens Health. 2016;61:242–8.

110. Maassen MS, Hendrix MJC, van Vugt HC, Veersma S, Smits F, Nijhuis JG. The choice of obstetric care by low-risk pregnant women in the Netherlands: implications for policy and management. Birth. 2008;35:277–82.

111. MacVicar J, Dobbie G, Owen-Johnstone L, Jgger C, Hopkins M, Kennedy J. Simulated home delivery in hospital: a randomised controlled trial. Br J Obstet Gynaecol. 1993;100:316–23.

112. Magann EF, Bronstein J, McKelvey SS, Wendel P, Smith DM, Lowery CL. Evolving trends in maternal fetal medicine referrals in a rural state using telemedicine. Arch Gynecol Obstet. 2012 Dec;286(6).

113. Mansbridge K. Nurse-to-nurse referral of patients in early pregnancy. Emerg Nurse. 2014;22(1):27–31.

114. Marlow N, Bennett C, Draper ES, Hennessy EM, Morgan AS, Costeloe KL. Perinatal outcomes for extremely preterm babies in relation to place of birth in England: the EPICure 2 study. Arch Dis Child - Fetal Neonatal Ed. 2014;99:F181–8.

115. Martijn L, Jacobs A, Amelink-Verburg M, Wentzel R, Buitendijk S, Wensing M. Adverse outcomes in maternity care for women with a low risk profile in The Netherlands: a case series analysis. BMC Pregnancy Childbirth. 2013;13:219.

116. Matthey S, Souter K, Mortimer K, Stephens C, Sheridan-Magro A. Routine antenatal maternal screening for current mental health: evaluation of a change in the use of the Edinburgh Depression Scale in clinical practice. Arch Womens Ment Health. 2016;19:367–72.

117. Mayer F, Bick D, Taylor C. Multidisciplinary care for pregnant women with cardiac disease: a mixed methods evaluation. Int J Nurs Stud. 2018;85:96–105.

118. McCool WF, Guidera M, Griffinger E, Sacan D. Closed claims analysis of medical malpractice lawsuits involving midwives: lessons learned regarding safe practices and the avoidance of litigation. J Midwifery Womens Health. 2015;60:437–44.

119. McIntyre MJ. Safety of non-medically led primary maternity care models: a critical review of the international literature. Aust Heal Rev. 2012;36(2):140–7.

120. McMurtrie J, Carling-Paul C, Teate A, Caplice S, Chapman M, Homer C. The St. George Homebirth Program: an evaluation of the first 100 booked women. Aust New Zeal J Obstet Gynaecol. 2009;49:631–6.

121. Mengel MB, Phillips WR. The quality of obstetric care in family practice: are family physicians as safe as obstetricians? J Fam Pract. 1987;24(2):159–64.

122. Meuli RL, Cohen LJ. Regionalization of perinatal care. West J Med. 1984;141(5):695–7.

123. Mito A, Arata N, Sakamoto N, Miyakoshi K, Waguri M, Osamura A, Kugishima Y, Metoki H, Yasuhi I. Present status of clinical care for postpartum patients with hypertensive disorders of pregnancy in Japan: findings from a nationwide questionnaire survey. Hypertens Pregnancy. 2015;34(2):209–20.

124. Monk A, Tracy M, Foureur M, Grigg C, Tracy S. Evaluating Midwifery Units (EMU): a prospective cohort study of freestanding midwifery units in New South Wales, Australia. BMJ Open. 2014;4:e006252.

125. Montgomery-Andersen RA, Willén H, Borup I. ‘There was no other way things could have been.’ Greenlandic women’s experiences of referral and transfer during pregnancy. Anthropol Med. 2010;17(3):301–13.

126. Morano S, Cerutti F, Mistrangelo E, Pastorino D, Benussi M, Costantini S, Ragni N. Outcomes of the first midwife-led birth centre in Italy: 5 years’ experience. Arch Gynecol Obstet. 2007;276:333–7.

127. Morley K. Epilepsy in pregnancy: the role of the midwife in risk management. Br J Midwifery. 2018 Sep;26(9):564–73.

128. Morriss FH. Increased risk of death among uninsured neonates. Health Serv Res. 2013;48(4):1232–55.

129. Morriss F. Interhospital transfers of maternal patients: cohort analysis of nationwide inpatient sample, 2011. Am J Perinatol. 2018;35:65–77.

130. Nuovo J. Clinical application of a high-risk scoring system on a family practice obstetric service. J Fam Pract. 1985;20(2):139–44.

131. Offerhaus PM, de Jonge A, van der Pal-de Bruin KM, Hukkelhoven CWPM, Scheepers PLH, Lagro-Janssen ALM. Change in primary midwife-led care in the Netherlands in 2000–2008: A descriptive study of caesarean sections and other interventions among 807,437 low risk births. Midwifery. 2015;31:648–54.

132. Offerhaus PM, Otten W, Boxem-Tiemessen JCG, de Jonge A, van der Pal-de Bruin KM, Scheepers PLH, Lagro-Janssen ALM. Variation in intrapartum referral rates in primary midwifery care in the Netherlands: a discrete choice experiment. Midwifery. 2015;31:e69–78.

133. Offerhaus PM, Geerts C, de Jonge A, Hukkelhoven CWPM, Twisk JWR, Lagro-Janssen ALM. Variation in referrals to secondary obstetrician-led care among primary midwifery care practices in the Netherlands: a nationwide cohort study. BMC Pregnancy Childbirth. 2015;15:42.

134. Papiernik E, Keith LG. The regionalization of perinatal care in France — description of a missing policy. Eur J Obstet Gynecol Reprod Biol. 1995;61:99–103.

135. Patterson J, Foureur M, Skinner J. Remote rural women’s choice of birthplace and transfer experiences in rural Otago and Southland New Zealand. Midwifery. 2017;52:49–56.

136. Peddle LJ, Brown H, Buckley J, Dixon W, Kaye J (MacDonald), Muise M, Rees E, Woodhams W, Young C. Voluntary regionalization and associated trends in perinatal care: The Nova Scotia Reproductive Care Program. Am J Obstet Gynecol. 1983;145(2):170–6.

137. Perdok H, Jans S, Verhoeven C, van Dillen J, Mol BW, de Jonge A. Intrapartum referral from primary to secondary care in the Netherlands: a retrospective cohort study on management of labor and outcomes. Birth. 2015;42(2):156–64.

138. Perdok H, Jans S, Verhoeven C, van Dillen J, Batenburg R, Mol BW, Schellevis F, de Jonge A. Opinions of professionals about integrating midwife- and obstetrician-led care in the Netherlands. Midwifery. 2016;37:9–18.

139. Persson G, Kern P. Reorganization of perinatal care in Greenland. Int J Circumpolar Health. 2004;63(sup2):397–8.

140. Phillippi JC, Holley SL, Thompson JL, Virostko K, Bennett K. A planning checklist for interprofessional consultations for women in midwifery care. J Midwifery Womens Health. 2019;64:98–103.

141. Posthumus AG, Borsboom GJ, Poeran J, Steegers EAP, Bonsel GJ. Geographical, ethnic and socio-economic differences in utilization of obstetric care in the Netherlands. PLoS One. 2016;11(6):e0156621.

142. Posthumus AG, Birnie E, van Veen MJ, Steegers EAP, Bonsel GJ. An antenatal prediction model for adverse birth outcomes in an urban population: the contribution of medical and non-medical risks. Midwifery. 2016;38:78–86.

143. Prentice A, Walton SM. Outcome of pregnancies referred to a general practitioner maternity unit in a district hospital. BMJ. 1989;299:1090–2.

144. Quinn EK, Noble J, Seale H, Ward JE. Provision of maternity care for women in remote Far West New South Wales: how far have we come? Aust J Rural Health. 2014;22:114–20.

145. Radomsky NA. Family practice obstetrics in a community hospital. Can Fam Physician. 1995;41:617–24.

146. Ravelli ACJ, Eskes M, Tromp M, Van Huis AM, Steegers EAP, Tamminga P, Bonsel GJ. Perinatale sterfte in Nederland gedurende 2000-2006; risicofactoren en risicoselectie [Perinatal mortality in The Netherlands 2000-2006; risk factors and risk selection]. Ned Tijdschr voor Geneeskd. 2008;152:2728–33.

147. Reddy K, Reginald P, Spring J, Nunn L, Mishra N. A free-standing low-risk maternity unit in the United Kingdom: does it have a role? J Obstet Gynaecol (Lahore). 2004;24(4):360–6.

148. Reilly SE, Brennecke SP, Smith J, Stewart MJ, Boland RA. Clinical features and outcomes of pregnancies complicated by pre-ecplampsia necessitating in-utero transfer. Pregnancy Hypertens. 2018;14:162–7.

149. Reither M, Germano E, DeGrazia M. Midwifery management of pregnant women who are obese. J Midwifery Womens Health. 2018;63:273–82.

150. Ressl B, O’Beirne M. Detecting breech presentation before labour: lessons from a low-risk maternity clinic. J Obstet Gynaecol Canada. 2015;37(8):702–6.

151. Reynolds J., Yudkin PL, Bull MJV. General practitioner obstetrics: does risk prediction work? J R Coll Gen Pract. 1988;38:307–10.

152. Richardson DK, Gabbe SG, Wind Y. Decision analysis of high-risk patient referral. Obstet Gynecol. 1984;63:496–501.

153. Richardson D, Rosoff A, Mcmenamin JP. Referral practices and health care costs: the dilemma of high risk obstetrics. J Leg Med. 1985;6(4):427–64.

154. Rijnders M, Baston H, Schönbeck Y, van der Pal K, Prins M, Green J, Buitendijk S. Perinatal factors related to negative or positive recall of birth experience in women 3 years postpartum in the Netherlands. Birth. 2008;35(2):107–16.

155. Roberts, Christine L, Henderson-Smart D, Ellwood DA, The High Risk Obstetric and Perinatal Advisory Working Group. Antenatal transfer of rural women to perinatal centres. Aust New Zeal J Obstet Gynaecol. 2000;40:377–84.

156. Rogers C, Pickersgill J, Palmer J, Broadbent M. Informing choices:outcomes for women at a stand-alone birth centre. Br J Midwifery. 2010;18(1):8–15.

157. Romijn A, Muijtjens AMM, de Bruijne MC, Donkers HHLM, Wagner C, de Groot CJM, Teunissen PW. What is normal progress in the first stage of labour? A vignette study of similarities and differences between midwives and obstetricians. Midwifery. 2016;41:104–9.

158. Rooks JP, Weatherby NL, Ernst EKM, Stapleton S, Rosen D, Rosenfield A. Outcomes of care in birth centers. The National Birth Center Study. N Engl J Med. 1989;321(26):1804–11.

159. Rooks JP, Weatherby NL, Ernst EKM. The National Birth Center Study part I — methodology and prenatal care and Rrferrals. J Nurse Midwifery. 1992;37(4):222–53.

160. Rooks JP, Weatherby NL, Ernst EKM. The National Birth Center Study part III — intrapartum and immediate postpartum and neonatal complications and transfers, postpartum and neonatal care, outcomes, and client satisfaction. J Nurse Midwifery. 1992;37(6):361–97.

161. Rosenblatt. Outcomes of regionalized perinatal care in Washington State. West J Med. 1988;149(1):98–102.

162. Rowe R, Fitzpatrick R, Hollowell J, Kurinczuk J. Transfers of women planning birth in midwifery units: data from the Birthplace prospective cohort study. BJOG. 2012;119:1081–90.

163. Rowe R, Li Y, Knight M, Brocklehurst P, Hollowell J. Maternal and perinatal outcomes in women planning vaginal birth after caesarean (VBAC) at home in England: secondary analysis of the Birthplace national prospective cohort study. BJOG. 2016;123:1123–32.

164. Ryan TD, Kidd GM. Maternal morbidity associated with in utero transfer. BMJ. 1989 Dec 2;299:1383–5.

165. Scherjon S. A comparison between the organization of obstetrics in Denmark and The Netherlands. Br J Obstet Gynaecol. 1986;93:684–9.

166. Scherman S, Smith J, Davidson M. The first year of a midwifery-led model of care in Far North Queensland. Med J Aust. 2008;188:85–8.

167. Schmidt N, Abelsen B, Oian P. Deliveries in maternity homes in Norway: results from a 2-year prospective study. Acta Obstet Gynecol Scand. 2002;81:731–7.

168. Schuit E, Hukkelhoven CWPM, van der Goes BY, Overbeeke I, Moons KGM, Mol BWJ, Groenwold RHH, Kwee A. Risk indicators for referral during labor from community midwife to gynecologist: a prospective cohort study. J Matern Neonatal Med. 2015;29(20):3304–11.

169. Schwartz RM, Muri JH, Overpeck MD, Pezzullo JC, Kogan MD. Use of high-technology care among women with high-risk pregnancies in the United States. Matern Child Health J. 2000;4(1):7–18.

170. Scupholme A, McLeod AGW, Robertson EG. A birth center affiliated with the tertiary care center: comparison of outcome. Obstet Gynecol. 1986;67(4):598–603.

171. Shaw R, Kitzinger C. Calls to a home birth helpline: empowerment in childbirth. Soc Sci Med. 2005;61:2374–83.

172. Shenai JP, Major CW, Gaylord MS, Blake WW, Simmons A, Oliver S, DeArmond D. A successful decade of regionalized perinatal care tennessee: the neonatal experience. J Perinatol. 1991;11(2):137–43.

173. Sidhu H, Heasley RN, Patterson CC, Halliday HL, Thompson W. Short term outcome in babies refused perinatal intensive care. BMJ. 1989 Sep 9;299:647–9.

174. Sloan EP, Kirsh S. Characteristics of obstetrical inpatients referred to a consultation-liaison psychiatry service in a tertiary-level university hospital. Arch Womens Ment Health. 2008;11:327–33.

175. Smit Y, Scherjon SA, Treffers PE. Elderly nulliparae in midwifery care in Amsterdam. Midwifery. 1997;13:73–7.

176. Smit Y, Scherjon S., Knuist M, Treffers P. Obstetric outcome of elderly low-risk nulliparae. Int J Gynecol Obstet. 1998;63:7–14.

177. Smit M, Ganzeboom A, Dawson JA, Walther FJ, Bustraan J, van Roosmalen JJM, te Pas AB. Feasibility of pulse oximetry for assessment of infants born in community based midwifery care. Midwifery. 2014;30:539–43.

178. Snowden JM, Tilden EL, Snyder J, Quigley B, Caughey AB, Cheng YW. Planned out-of-hospital birth and birth outcomes. N Engl J Med. 2015;373(27):2642–53.

179. Stern C, Permezel M, Petterson C, Lawson J, Eggers T, Kloss M. The Royal Women’s Hospital Family Birth Centre: the first 10 years reviewed. Aust New Zeal J Obstet Gynaecol. 1992;32(4):291–6.

180. Stewart MJ, Smith J, Boland RA. Optimizing outcomes in regionalized perinatal care: integrating maternal and neonatal emergency referral, triage, and transport. Curr Treat Options Pediatr. 2017;3:313–26.

181. Stolp I, Smit M, Luxemburg S, van den Akker T, de Waard J, van Roosmalen J, de Vos R. Ambulance transfer in case of postpartum hemorrhage after birth in primary midwifery care in the Netherlands: a prospective cohort study. Birth. 2015;42(3):227–34.

182. Street P, Gannon MJ, Holt EM. Community obstetric in West Berkshire. BMJ. 1991;302:698–700.

183. Strobino DM, Beth Silver G, Allston AA, Grason HA. Local health department perspectives on linkages among birthing hospitals. J Perinatol. 2003;23:610–9.

184. Styles M, Cheyne H, O’Carroll R, Greig F, Dagge-Bell F, Niven C. The Scottish Trial of Refer or Keep (the STORK study): midwives’intrapartum decision making. Midwifery. 2011;27:104–11.

185. Sullivan NH, Witte M. Care of the at-risk neonate born at home — a model for nurse-midwife/physician collaboration. J Nurse Midwifery. 1995;40(6):534–40.

186. Suzuki S, Satomi M, Miyake H. Referrals during labor in midwifery care. J Nippon Med Sch. 2009;74(4):226–8.

187. Suzuki S. Trend analysis of primary midwife-led delivery care at a Japanese perinatal center. Int J Med Sci. 2014;11:466–70.

188. Symon A, Winter C, Donnan PT, Kirkham M. Examining autonomy’s boundaries: a follow-up review of perinatal mortality cases in UK independent midwifery. Birth. 2010;37(4):280–7.

189. Tilyard MW, Seddon RJ, Oakley W, Murdoch CJ. Is outcome for general practitioner obstetricians influenced by workload and locality? N Z Med J. 1988 Apr 27;101:207–9.

190. Tromp M, Eskes M, Reitsma JB, Erwich JJHM, Brouwers HAA, Rijninks-van Driel GC, Bonsel GJ, Ravelli ACJ. Regional perinatal mortality differences in the Netherlands; care is the question. BMC Public Health. 2009 Dec 14;9:102.

191. Tucker J. Guidelines and management of mild hypertensive conditions in pregnancy in rural general practices in Scotland: issues of appropriateness and access. Qual Saf Heal Care. 2003;12:286–90.

192. Tucker J, McVicar A, Pitchforth E, Farmer J, Bryers H. Maternity care models in a remote and rural network: assessing clinical appropriateness and outcome indicators. Qual Saf Heal Care. 2010;19:83–9.

193. Van Alten D, Eskes E, Treffers PE. Midwifery in the Netherlands. The Wormerveer study; selection, mode of delivery, perinatal mortality and infant morbidity. BJOG. 1989;96:656–62.

194. van der Kooy J, de Graaf JP, Birnie DE, Denktas S, Steegers EAP, Bonsel GJ. Different settings of place of midwife-led birth: evaluation of a midwife-led birth centre. Springerplus. 2016;5:786.

195. van Haaren KMA, Springer MP. De kwaliteit van het verloskundig handelen van de huisarts [The quality of obstetric care given by the general practitioner]. Huisarts Wet. 2002;45(11):586–91.

196. Van Otterloo LR, Connelly CD. Risk-appropriate care to improve practice and birth outcomes. J Obstet Gynecol Neonatal Nurs. 2018;47:661–72.

197. van Stenus CMV, Gotink M, Boere-Boonekamp MM, Sools A, Need A. Through the client’s eyes: using narratives to explore experiences of care transfers during pregnancy, childbirth, and the neonatal period. BMC Pregnancy Childbirth. 2017;17:182.

198. van Stenus CMV, Boere-Boonekamp MM, Kerkhof EFGM, Need A. Client experiences with perinatal healthcare for high-risk and low-risk women. Women and Birth. 2018;31:e380–8.

199. van Wagner V, Osepchook C, Harney E, Crosbie C, Tulugak M. Remote midwifery in Nunavik, Québec, Canada: outcomes of perinatal care for the Inuulitsivik health centre, 2000-2007. Birth. 2012 Sep;39(3):230–7.

200. Vause S, Clarke B. Risk stratification and hierarchy of antenatal care. Best Pract Res Clin Obstet Gynaecol. 2014;28:483–94.

201. Vedam S, Goff M, Marnin VN. Closing the theory–practice gap: intrapartum midwifery management of planned homebirths. J Midwifery Womens Health. 2007;52(3):291–300.

202. Viisainen K, Gissler M, Hemminki E. Birth outcomes by level of obstetric care in Finland: a catchment area based analysis. J Epidemiol Community Heal. 1994;48:400–5.

203. Vos AA, van Voorst SF, Posthumus AG, Waelput AJM, Denktaş S, Steegers EAP. Process evaluation of the implementation of scorecard-based antenatal risk assessment, care pathways and interdisciplinary consultation: the Healthy Pregnancy 4 All study. Public Health. 2017;150:112–20.

204. Waldenström U, Nilsson C-A. A randomized controlled study of birth center care versus standard maternity care: effects on women’s health. Birth. 1997;24(1):17–26.

205. Waldenström U, Nilsson C-A, Winbladh B. The Stockholm Birth Centre Trial: maternal and infant outcome. Br J Obstet Gynaecol. 1997;104:410–8.

206. Wallace EM, Mackintosh CL, Brownlee M, Laidlaw L, Johnstone FD. A study of midwife-medical staff interaction in a labour ward environment. J Obstet Gynaecol (Lahore). 1995;15(3):165–70.

207. Wiegers TA, van der Zee J, Keirse MJNC. Transfer from home to hospital: what is its effect on the experience of childbirth? Birth. 1998;25(1):19–24.

208. Woodcock HC, Read AW, Moore DJ, Stanely FJ, Bower C. Planned homebirths in Western Australia 1981-1987: a descriptive study. Med J Aust. 1990 Dec;153:672–8.

209. Woodhart L, Goldstone J, Hartz D. The stories of women who are transferred due to threat of preterm birth. Women and Birth. 2018;31:307–12.

210. Wright JD, Silver RM, Bonanno C, Gaddipati S, Lu Y-S, Simpson LL, Herzog TJ, Schulkin J, D’Alton ME. Practice patterns and knowledge of obstetricians and gynecologists regarding placenta accreta. J Matern Neonatal Med. 2013;26(16):1602–9.
