# Supplementary material for: Towards a better understanding of risk selection in maternal and newborn care: A systematic scoping review
Source: PLoS One. 2020 Jun 8;15(6):e0234252. doi: 10.1371/journal.pone.0234252 (PMC7279596; doi:10.1371/journal.pone.0234252)
Supplement: Attachment — Submitted filename: Scop Rev Risk Selec - Response to Reviewers.docx [file pone.0234252.s006.docx]

Bahareh Goodarzi

Department of Midwifery Science

Amsterdam UMC location VUmc

Medical Faculty Room G-102D

Van der Boechorststraat 7, 1081 BT Amsterdam
T: 020-444 54 79

E: [b.goodarzi@amsterdamumc.nl](mailto:b.goodarzi@amsterdamumc.nl)

Amsterdam, 24-01-2020

Dear Dr. Portela,

Thank you for considering our manuscript for publication in *PLOS ONE*. We appreciate your feedback.

We have discussed your feedback among our team and seriously considered your comments, which has resulted in revisions of the manuscript.

We agree that additional details about the included papers and our analysis can provide the reviewers and the readers further insight into how we arrived at our results. Therefore, following your advice, we have extended the S3 table (now S4) with further details of the papers, including information about papers’ study objective and study design, and the identified main category. We, furthermore, have added a list of identified themes and categories (now S3).

We look forward to your reply.

Yours sincerely, also on behalf of the rest of the research team,

Bahareh Goodarzi
